# Multi-criteria suitability analysis for neglected and underutilised crop species in South Africa

**DOI:** 10.1371/journal.pone.0244734

**Published:** 2021-01-19

**Authors:** Hillary Mugiyo, Vimbayi G. P. Chimonyo, Mbulisi Sibanda, Richard Kunz, Luxon Nhamo, Cecelia R. Masemola, Caroline Dalin, Albert T. Modi, Tafadzwa Mabhaudhi

**Affiliations:** 1 Centre for Transformative Agricultural and Food Systems, School of Agricultural, Earth & Environmental Sciences, University of KwaZulu-Natal, Pietermaritzburg, South Africa; 2 Centre for Water Resources Research, School of Agricultural, Earth and Environmental Sciences, University of KwaZulu-Natal, Scottsville, South Africa; 3 Water Research Commission of South Africa, Pretoria, South Africa; 4 Institute for Sustainable Resources, University College London, London, United Kingdom; Potsdam Institute for Climate Impact Research, GERMANY

## Abstract

Several neglected and underutilised species (NUS) provide solutions to climate change and creating a Zero Hunger world, the Sustainable Development Goal 2. Several NUS are drought and heat stress-tolerant, making them ideal for improving marginalised cropping systems in drought-prone areas. However, owing to their status as NUS, current crop suitability maps do not include them as part of the crop choices. This study aimed to develop land suitability maps for selected NUS [sorghum, (*Sorghum bicolor*), cowpea (*Vigna unguiculata*), amaranth and taro (*Colocasia esculenta*)] using Analytic Hierarchy Process (AHP) in ArcGIS. Multidisciplinary factors from climatic, soil and landscape, socio-economic and technical indicators overlaid using Weighted Overlay Analysis. Validation was done through field visits, and area under the curve (AUC) was used to measure AHP model performance. The results indicated that sorghum was highly suitable (S1) = 2%, moderately suitable (S2) = 61%, marginally suitable (S3) = 33%, and unsuitable (N1) = 4%, cowpea S1 = 3%, S2 = 56%, S3 = 39%, N1 = 2%, amaranth S1 = 8%, S2 = 81%, S3 = 11%, and taro S1 = 0.4%, S2 = 28%, S3 = 64%, N1 = 7%, of calculated arable land of SA (12 655 859 ha). Overall, the validation showed that the mapping exercises exhibited a high degree of accuracies (i.e. sorghum AUC = 0.87, cowpea AUC = 0.88, amaranth AUC = 0.95 and taro AUC = 0.82). Rainfall was the most critical variable and criteria with the highest impact on land suitability of the NUS. Results of this study suggest that South Africa has a huge potential for NUS production. The maps developed can contribute to evidence-based and site-specific recommendations for NUS and their mainstreaming. Also, the maps can be used to design appropriate production guidelines and to support existing policy frameworks which advocate for sustainable intensification of marginalised cropping systems through increased crop diversity and the use of stress-tolerant food crops.

## 1. Introduction

The world is challenged by the need to feed a growing population with healthy food while minimising the negative impacts on the environment and adapting to changing climate [1]. Despite the importance of smallholder agriculture to global food production and poverty reduction [2], there has been a decline in the level of agricultural production in the Sub Saharan Africa (SSA) region [3]. More so in South Africa (SA), the contribution of agriculture to household food consumption among smallholder farmers continues to fall [1]. It is understood that inherent water scarcity, exacerbated by climate variability and changes in land use, has contributed to reduced land available for agricultural expansion for the production of major crops especially in resource-poor farming systems [4]. Considering these challenges, agriculture requires innovative approaches that seek to address, not only issues of food and nutrition security but also environmental degradation, adapt to climate variability and land use planning. Sustainable intensification of smallholder food production systems is considered essential to meeting the United Nations Sustainable Development Goal 1 (poverty eradication) and 2 (zero hunger) [5]. There is a need to introduce and promote practices that fit “into” or “with” current smallholder production systems while complementing existing efforts to improve resilience to climate variability and change as well as intensifying productivity for sustainable food and nutrition security [6].

Neglected and underutilised crop species are an option for redressing food and nutrition challenges faced in marginalised communities [7]. These crops are native to specific areas in geological time [8] and are known to be suitable in marginal areas characterised by severe dry spells and flash floods [9]. Across the world, several research initiatives examined the mechanisms that allow for stress adaptation within a range of NUS [10–12]. For instance, in SA Chibarabada et al [10] modelled productivity of ground nuts under water deficit conditions, in Malaysia Peter et al. [11] examined the adoption of underutilised crops, while Ebert [12] from Taiwan, assessed the potential of underutilized traditional vegetables and legume crops in contributing to food and nutritional security. These studies illustrate that, while NUS may be well adapted to multiple stress conditions, they are grown in geographical pockets that are often far from where they could provide the most positive contribution to food and nutrition security [9]. The lack of scientific evidence has resulted in the slow promotion of NUS into existing food systems, be it formal or informal [13]. As such, policy frameworks on agriculture, health and environment continue to remain silent on the potential use of NUS in contributing towards increasing adaptation of marginalised agricultural systems to climate risks. In addition, little mentioned about their contribution towards good health as well as nutrition and rehabilitation of degraded agricultural lands. As such, information detailing the suitability of NUS is essential if they are to be recognised as a sustainable and plausible option for contributing towards the sustainable development and improved resilience of marginalised farming communities [14].

Land suitability analysis assesses the appropriateness of crops to a specific practice or land use [15]. Specifically, land suitability evaluates land capability as well as other factors such as land quality, land ownership, customers demand, economic values and proximity to different accesses [16]. Multi-criteria decision making (MCDM), also referred to as, Multi-criteria decision analysis (MCDA) can be used to define land potential to solve complex problems of land-use and land-use changes [17–19]. Multi-criteria decision-analysis methodologies can overcome problems related to vagueness in definition and other uncertainties, especially in the context of NUS suitability analysis [20]. Land suitability analysis can be done by using geographic information system based MCDM to identify suitable areas for cultivating NUS. To improve the interpretations of MCDA, Saaty (1980) introduced the Analytic Hierarchy Process (AHP) as a method to capture aspects of a decision in both a subjective and objective manner to reduce confounding [21, 22]. The AHP methodology provides scope for combining expert opinions with numerical predictions from biophysical models to provide an integrated approach to resource management [23, 24]. Similar techniques have been used in agriculture by Musakwa identifying land suitable for agricultural land reform [25], Kazemi et al. [26] for rain fed wheat, Zabihi et al. [27] for citrus, Kihoro et al. [28] for rice in Kenya, Benke and Pelizaro [29] for wheat and rye-grass production.

Currently, the delineation of South Africa’s rain-fed agricultural land use is for few major cash crops such as maize, sugar cane, and soybean. The few crops reflect the current lack of agro-biodiversity, which culminates in increased sensitivity of agriculture to climate risks [30]. An example is the 2015/16 ENSO drought that caused South Africa to import more than 30% of its annual cereal grain requirements due to poor harvests. In general, NUS are hypothesised to be suitable for marginal agro-ecologies [6] and can help increase the resilience of rain-fed cropping systems in the wake of climate variability and change. In this regard, NUS can offer ecologically viable options for increasing agriculture productivity, especially in marginal areas, as they are locally adapted and would not strain the environment further [31]. Therefore, the promotion of indigenous crops such as sorghum-*Sorghum bicolor*, cowpea-*Vigna unguiculata* and taro-*Colocasia esculenta* is integral to ensuring that households consume more diverse diets [32]. Mapping NUS production potential zones in SA, will help inform decision on where NUS can be promoted as part of the crop choice, assist decision-makers in formulating policies with a sustainable intensification concept and then the creation of markets for NUS, which will enhance food and nutrition security. Therefore, the main objective of the research is to identify potential areas suitable for sorghum-, cowpea, taro, and amaranth- using a GIS-based MCDA-AHP.

## 2. Methodology

### 2.1 Multi-criteria decision analysis (MCDA) approach

Crop suitability is a function of crop requirements and land characteristics, therefore matching the land characteristics with the crop requirements gives the suitability [33]. Suitability analysis has to be carried out in such a way that farming systems and local needs are reflected well in the final decisions [34]. The MCDA combines qualitative and quantitative criteria while specifying the degree and nature of the relationships between those criteria to support spatial decision-making [35].

**Fig 1:**
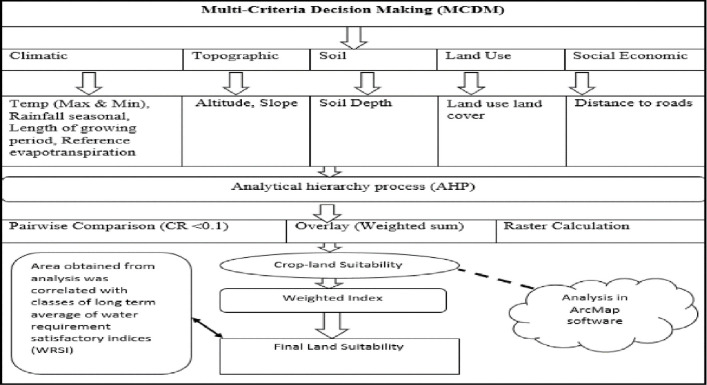
Framework used in computing suitability indices for neglected and underutilised crop species in South Africa (developed by the authors).

The process of evaluating the suitability of land for a specific purpose requires a comprehensive analysis of natural factors and the socio-economic factors which influence the land [36, 37]. The elements used can be divided into high and lower factors based on experts’ opinion weights [27]. High-level factors in crop suitability analysis are natural or biophysical factors that directly affect the growth of crops, for example, rainfall, and temperature and soil fertility. The lower level factors are social and economic factors which indirectly affect crop growth, but influence land use degree of appropriateness to a purpose [38]. The interactions, dependencies and feedback between higher and lower-level elements form a multi-criteria land evaluation approach for a sustainable NUS production. Fig 1 presents a conceptual framework for developing NUS Cropland suitability maps using GIS.

**The general land use suitability model is:**

$$S(a1\ldots ,an)=\sum_{j=1}^{n}wjbj.$$ (1)

where S (a_1…,_ a_n_) is suitability measure, and b_j_ is the j^th^ largest of the a_1_ factors affecting the suitability of the sites [21, 39]. A weighted average is an average where each observation in the data set is multiplied by a predetermined weight before calculation Eq (1) [40]. The ordered weighted averaging (OWA) operator is a non-linear operator as a result of the process of determining the *b*_*j*_, and this was achieved by choosing different weights to implement different aggregation operators’ Eq (1).

## 2.2 Data sources

For this study, data were obtained from the South African Quaternary Catchments database (Table 1). The multidisciplinary data was grouped into climatic, soil and landscape attributes, social-economic and technical indicators. Nine parameters were used, and these included five climatic, three soil, and one social parameter (Table 1). High-resolution climatic parameters were derived from 1950 to 2000, a 50-year time series of continuous daily data from selected 1 946 stations Quaternary Catchments covering South Africa [41]. The datasets were developed by the Water Research Commission funded in a project titled “Mapping the Mean Annual Precipitation and Other Rainfall Statistics” [42]. The spatial resolution of climatic data was one arc minute; this implies that one grid is represented as 1.7 x 1.7 km. Lynch (2004) calculated monthly precipitation by using a geographically weighted regression method, and monthly means of daily average temperatures were derived from [43]. Abrams [44] indicated that over 70% of South African food production is rain-fed. In South Africa only 1,5% of the land is under irrigation, producing approximately 30% of the country’s crops. Therefore, all climatic parameters were calculated using seasonal and not annual data. Wet periods can be calculated from daily precipitation events like the start of the season, dry spells, end of the season. In SA, precipitation is undoubtedly the dominating factor determining crop production, especially in marginal areas where irrigation facilities are limited for smallholder farmers [45].

**Table 1:** Factors used to delineate land suitability maps for neglected and underutilised crop species.

Factors	Source
Climate-related factors	
Precipitation (mm) 1.7 km resolution	South African Quaternary Catchments database- Water Research Commission
Temperature 1.7 km resolution	South African Quaternary Catchments database- Water Research Commission
Reference crop evapotranspiration (ETo) millimetres (mm) or (lm^-2^) 1.7 km resolution	South African Quaternary Catchments database- Water Research Commission
Length of growing period (LGP) 1.7 km resolution	South African Quaternary Catchments database- Water Research Commission
Water Requirement Satisfaction Index (WRSI)-at 10 km resolution	Fewsnet https://earlywarning.usgs.gov/fews
**Soil and landscape attributes used to delineate land suitability maps for neglected and underutilised crop species**	
Soil depth at 250 m resolution	South African Quaternary Catchments database- Water Research Commission
Elevation (mm) 30 m resolution	https://earthexplorer.usgs.gov/
Slope	South African Quaternary Catchments database- Water Research Commission
**Social and economic factors used to delineate land suitability maps for NUS.**	
Distance from road/accessibility	South African Quaternary Catchments database- Water Research Commission

A full description of each parameter are explained in the S1 Appendix.

All thematic variables used in this study were converted to raster layers. Before the analysis, all thematic layers were resampled into the World Geodetic System 1984 (WGS84) geo-referencing system [46]. The resolution of finer grid layers was resampled to 1.7 km resolution of climatic factors. All the transformations of the GIS layers were done in ArcGIS.

### 2.3 Analytic hierarchy process (AHP)

The analytic hierarchy process (AHP) is the most widely accepted method and is considered by many as the most robust of MCDA [47]. The AHP helps to capture both subjective and objective aspects of a decision by reducing complex decisions to a series of pairwise comparisons and then synthesising the results [17]. Since AHP considers a set of evaluation criteria and a set of alternative options among which the best decision is to be made, a 9-point scale measurement was used in this study (Table 2). In this study, the AHP calculator was used to calculate weights [48]. The assignments of weights were based on information from literature, as well as the team’s local knowledge and expert consultation (soil scientist, GIS and remote sensing specialists from the University of KwaZulu Natal) (Table 3).

**Table 2:** The fundamentals for pairwise comparison [49].

Intensity of importance	Definition	Explanation
1	equal importance	Two activities contribute equally to the objective
3	moderate importance of one over another	Experience and judgment slightly favour one activity over another
5	the strong or essential importance	Experience and judgment strongly favour one activity over another
7	very strong or demonstrated importance	Activity is strongly favoured, and its dominance showed in practice
9	extreme importance	The evidence favouring one activity over another is of the highest possible order of affirmation
2,4,6 and 8	Even numbers represent intermediate values between the two adjacent judgement	When compromise is needed

Factor weights were calculated by comparing two factors together at a time. The AHP weights were calculated using Microsoft Excel. Table 3 shows a pairwise comparison matrix for the research.

**Table 3:** Pairwise comparison matrix.

Factors	Rainfall	Temp	ET_o_	LGP	Elevation	Slope	LULC	Soil Depth	Distance to Road	Weight
Rainfall	1	2	2	2	5	5	3	2	9	0.24
Temp	1/2	1	2	3	3	3	3	2	8	0.18
ET_o_	1/2	1/2	1	1/3	5	3	3	2	5	0.13
LGP	1/2	1/3	3	1	5	3	3	3	5	0.17
Elevation	1/5	1/3	1/5	1/5	1	2	1/2	1/2	2	0.04
Slope	1/5	1/3	1/3	1/3	2	1	2	2	5	0.08
LULC	1/3	1/3	1/3	1/3	2	1/2	1	1/2	3	0.06
Soil Depth	1/2	1/2	1/2	1/3	2	1/2	2	1	5	0.08
Distance from Road	1/9	1/8	1/5	1/5	1/2	1/5	1/3	1/5	1	0.02

Maximum eigenvalue (λmax) = 9.6082, n = 9, Consistency index (CI) = (λmax−n)/(n−1) = 0.07602, Random index (RI) = 1.45, Consistency Ratio (CR) = CI/RI = 0.052428.

The pairwise comparisons in the AHP were determined according to the scale introduced by Saaty [50], with values from 9 to 1/9. A rating of 9 indicates that concerning the column factor, the row factor is more important. On the other hand, a rating of 1/9 indicates that relative to the column factor, the row factor is less important. In cases where the column and row factors are equally important, they have a rating value of 1. Through the pairwise comparison matrix, the AHP calculates the weighting for each criterion by taking the Eigenvector corresponding to the largest Eigenvalue of the matrix and then normalising the sum of the components to unity [51]. The ratio scales were derived from the principal Eigenvectors, and the consistency index was derived from the principal Eigenvalue. An eigenvalue is a number, which explains how much variance is spread out [52]. According to Brandt et al. [53] and Feng et al. [54], the AHP has a limitation of coming up with weights; it is subjective. The inconsistency can be improved by:

- Deriving pairwise matrix based on a scientific objective in non-scare data situation [55] (Table 3),
- Estimating the relative importance of factors individually and based more on scientists' opinion through informal interviews with key informants like a ministry of Agriculture[56] and
- Giving attention to an upper limit, the upper limit is a consistency ratio (CR) that must be less than 0.1 for a pairwise matrix judgment to be accepted [57]. To minimise the interrelationship among various factors included in the AHP approach, data reduction method such as Ordered Weighted Averaging (OWA) was used [58]. The weighted linear combination allows the variability of continuous and discrete factors to be retained and standardised to a standard numeric range [21].

#### 2.3.1 Fitting neglected and underutilised crop species in ecophysiology based on drought-tolerance characteristics

Agro-climatic indices were calculated to estimate phenological phases of crops to fit NUS in an environment (Table 4). The dynamic consideration of crop phenology allows assessing effects of agro-climate-factors to phenological development of NUS. The overall suitability was estimated based on Liebig's law of the minimum [59]. The Liebig’s law of the minimum to provide a flexible framework to assess climate suitability of crops in a situation where the crop suitability is subjected to imprecision and vagueness, or the pairwise comparisons are subjective especially when fuzzy AHP was used to classify NUS [26, 60]. It is based on three types of mathematical functions; the equations transform each variable to a suitability value varying from 1 (unsuitable) to 1 (optimum or highly suitable). Liebig’s law of the minimum is the outcome of AHP using the minimum t-norm between variables [61]. The mathematical expression for this type of relationship was formulated as follows.

$$S(V)=\begin{array}{c} 0\\ \frac{\left\{V-V\min\right\}}{\left\{\mathrm{Vol}-V\min\right\}}\\ 1 \end{array}if\;V\leq \mathrm{Vmin};\;\mathrm{if}\;\mathrm{Vmin}<V<\mathrm{Vol};\;\mathrm{if}\;V\geq \mathrm{Vol}$$ (2)

where S (V) is the suitability index as a function of the individual variable; V is the parameter; V_min_ indicates the minimum value of V required for crop growth; Vol is the lowest optimum value of V at or beyond which the highest suitability can be obtained.

In general, an increase in precipitation increases the suitability of crop in semi-arid regions. Based on the water use of a crop, the lower limit of precipitation was used to delineate area suitable for a crop, for example, 111 mm per year was used for amaranth (Table 4). According to FAO, a minimum of 500 mm rainfall per year is required to achieve reasonable economic yields, therefore, we used 500 mm as the upper threshold in our stepwise function [62]. Some variable like the terrain is inversely correlated with growth suitability (Table 4); the following criterion was used to mark the suitability of NUS.

$$S(V)=\frac{\begin{array}{c} 1\\ \left\{Vmax-\;V\right\} \end{array}}{\begin{array}{c} \left\{Vmax-Vou\right\}\\ 0 \end{array}}if\;V\leq Vou;\;if\;Vou<V<Vmax;\;if\;V\geq Vmax$$ (3)

where V_max_ is the maximum value of variable V beyond which no cropping is possible; Vou is the uppermost optimum value of V for cropping. In all areas with 0 to 5% slope has no limitation about the steepness and above 5%- optimal upper bound (Vou) field tends to have challenges in using have machines.

**Table 4:** Characteristics of sorghum [63], cowpea [63], taro [64] and amaranth [65].

	Sorghum (*Sorghum bicolor*)	Cowpea (*Vigna unguiculata*)	Taro (*Colocasia esculenta*)	Amaranth- (*Amaranthu*s)
Water use (mm)	261–415	133–265	800–1 288	111–448
Precipitation per season (mm)	450–800	400–700	800–2000	400–650
Time to maturity (Days)	100–120	90–150	240–300	20–45
Temperature range (°C)	26–30	25–30	25–32	18–30
Yield (kg ha^-1^)	2802–4 304	776–1 120	3 830–17 330	*3 400–5 200*

### 2.4 Qualitative land suitability classification

In this study, five different classes from FAO land suitability framework were used to quantify the magnitude of suitability for NUS within South Africa (Table 5). It classified the land into four suitability classes: land suitability orders, land suitability classes, land suitability sub-classes and land suitability units [66]. In FAO, orders indicate lands suitable for crops (S) or not suitable for crops (N) while classes show the degree of land suitability, such as (S1) highly suitable, (S2) moderately suitable, (S3) marginally not suitable, (N1) currently not suitable and (N2) permanently not suitable, and then subclass explains limitations. The classification designates a single index of use as best on each land unit [67].

**Table 5:** Suitability indices for the different suitability classes [68].

Suitability Class	Suitability index (SI)	Description Class
S1	Highly suitable (>80)	Land having no limitations for a given use, or constraints that do not reduce the productivity and benefits appreciably, with no need for a high level of input
S2	Moderately suitable (60–80)	Land having minor limitations that could reduce productivity or benefits, additive inputs are required to reach the same yield as that of class S1
S3	Marginally suitable (45–59)	Land having moderate limitations for a particular use, in which the amount of surplus input is only marginally justified
N1	Currently unsuitable (30–44)	Land with severe limitations for land use under consideration. Every sustainable use is precluded, and the costs for correction are unacceptable with the existing condition. Only new technologies could improve land productivity
N2	Permanently unsuitable (<30)	Land-use type under analysis is not acceptable at all for the land.

### 2.5 Validation of cropland suitability

The validation data was gathered through field surveys in one KwaZulu Natal conducted between 1^st^ of October to 21^st^ of November 2019. A total of 60 GPS locations of taro, amaranth, sorghum and cowpea were randomly collected during the survey. The GPS locations were measured at the centre of an identified field. The GPS locations were captured in excel and GPS locations were converted to a point map in a GIS. The crop presence was captured as one of the attribute tables. A total of 600 points were randomly generated in a GIS across South Africa. These points were used to represent the absence (value 0) of the crops. We used a ratio of 1:10 between known present points to pseudo-absence; hence, 600 pseudo absence points were generated [69]. These two point maps were merged into one layer, which was then overlaid with the MCDM/AHP derived suitability maps. A new table containing the presence and absence as well as the crop suitability information was produced and exported as an excel spreadsheet. This data was then used to measure the magnitude of agreement between the generated NUS suitability maps, and the field measured locations of crops using the receiver operating characteristic (ROC), and the area under the curve (AUC) derived based on the logistic regression. Each crops accuracy assessment using the logistic regression analysis was carried out in the statistical package R version 3.6.1 [70] using the ‘RATTLE’ library [71]. The ROC plot has an x-axis indicating the false-positive error rate, which signifies a wrong prediction by the model. The y-axis shows the positive rate, indicating a correct prediction by the model [71]. An AUC value that is less than or equal to 0.5 indicates a random prediction, while AUC values higher than 0.5 and closer to 1 indicates a better prediction by the model [72, 73]. The composite operator helps illustrate how well two layers or maps agree in terms of how the categories are clustered spatially.

We further checked the magnitude of dryness of classes of a correlation analysis between the general NUS land suitability index and the mean average of Water Requirement Satisfaction Index (WRSI) from 1981 to 2017 from Famine Early Warning Systems Network (FEWSNET) was used to compare the results. Water Requirement Satisfaction Index was developed by the FAO and mostly used by FEWSNET to monitor and investigate crop production in agricultural drought-prone parts of the world. The WRSI is an indicator of crop performance based on the availability of water to the crop during a growing season [74]. The classes of WRSI are crop failure- less 49%, Poor-50-79%, average-80-94, and good-95-100%.

## 3. Results

### 3.1 Sorghum land suitability map

**Fig 2:**
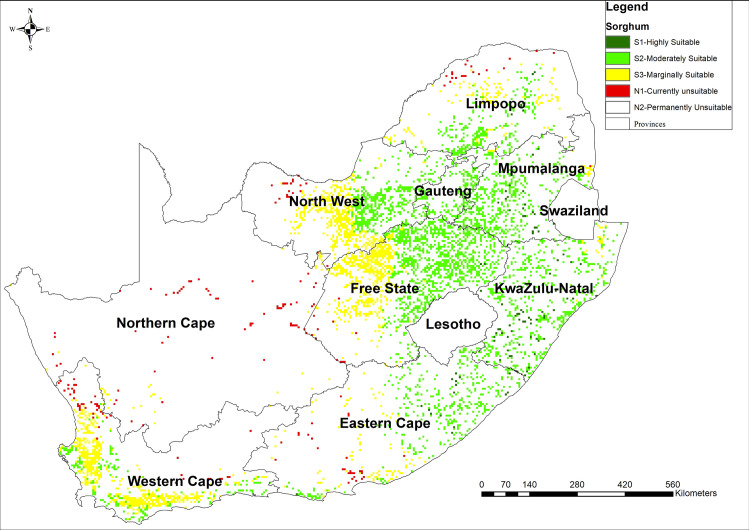
Suitability map for sorghum production in South Africa computed using MCDA-AHP and OWA operators [Source, *South African Quaternary Catchments database*, *(*https://doi.org/10.6084/m9.figshare.13179881*)*, *in ArcGIS 10*.*5]*.

Fig 2 presents the results of the analysis of the suitability of sorghum-based on MCDA-AHP and OWA operators. These results show the existing distribution of the land suitability classes, excluding areas where present land use is nature conservation, plantation, urban and water. Results indicated that there is about 2% of land that is highly suitable (S1) for the production of sorghum. Moderately suitable (S2) land constitutes the most substantial proportion (61%) of the calculated arable land of South Africa (12 655 859 ha) while marginally suitable (S3) and unsuitable (N1) constitutes 33% and 4%, respectively of calculated arable land (Fig 2). Large areas suitable (S1 and S2) land were concentrated in eastern provinces and suitability intensity decrease towards western provinces (Fig 2). A total of 60 GPS location was used to confirm the presence of sorghum within selected locations in KwaZulu Natal province.

### 3.2 Cowpea land suitability map

**Fig 3:**
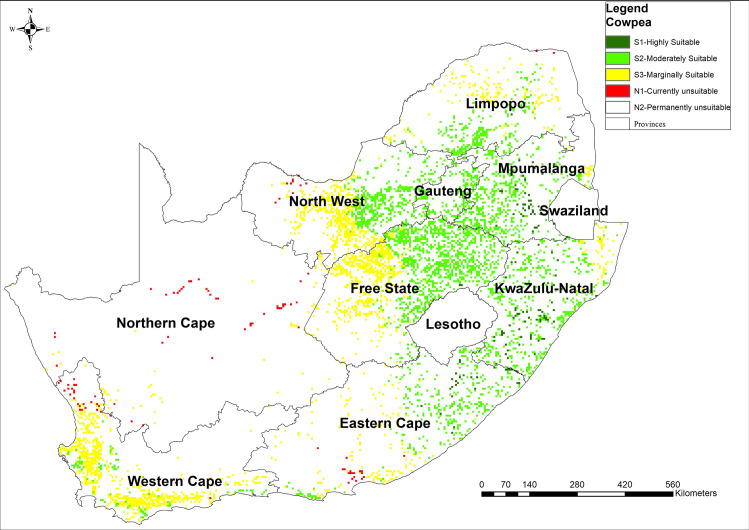
Suitability map for cowpea production in South Africa computed using MCDA-AHP and OWA operators [Source, *South African Quaternary Catchments database*, *(*https://doi.org/10.6084/m9.figshare.13179881*)*, *in ArcGIS 10*.*5]*.

Cowpea suitability varies across the country. The results indicated that there is about 3% of the land that is highly suitable (S1) for the production of cowpea. Moderately suitable (S2) land constitutes the most substantial proportion with 56% of the calculated arable land of South Africa (12 655 859 ha) while marginally suitable (S3) and unsuitable (N1) constitutes 39% and 2%, respectively of calculated arable land (Fig 3). The spatial suitability is high in south-eastern provinces and central provinces of South Africa. The intensity of suitability decreases from the central part of the country to the western regions of the country (Fig 3). Similar to sorghum, the distribution of suitability was consistent, but not in the order with rainfall, slope, soil depth and ET_O_ distribution.

### 3.3 Taro land suitability map

**Fig 4:**
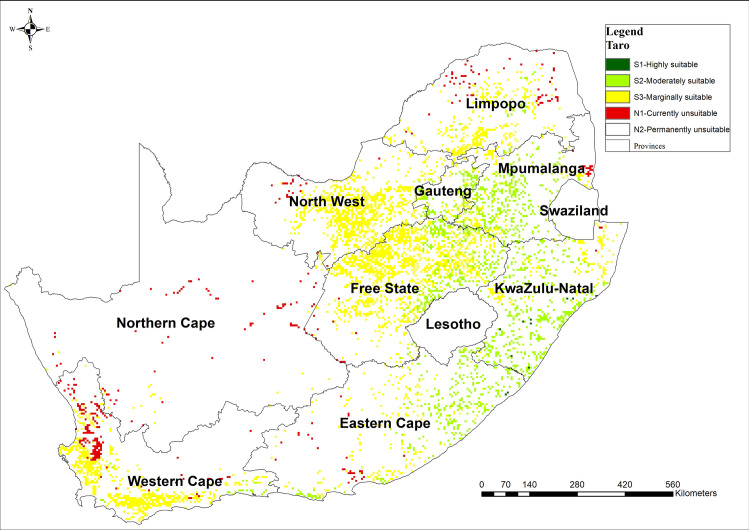
Suitability map for taro production in South Africa computed using MCDA-AHP and OWA operators [Source, *South African Quaternary Catchments database*, *(*https://doi.org/10.6084/m9.figshare.13179881*)*, *in ArcGIS 10*.*5]*.

Fig 4 presents the spatial distribution of the suitability scores for taro-based on MCDA-AHP method. The results indicated that there is about 0.4% of the land that is highly suitable (S1) for the production of taro. Moderately suitable (S2) land constitutes 28% of the calculated arable land of South Africa (12 655 859 ha) while marginally suitable (S3) constitutes the most substantial proportion 64% and (N1) 7% of calculated arable land. Taro suitability is high in KwaZulu Natal and Mpumalanga provinces. Limpopo, North West, Northern Cape and Western Cape are marginally suitable for taro (Fig 4). The distribution of taro suitability was consistent maximum temperature and length of the growing season and rainfall distribution.

### 3.4 Amaranth suitability

**Fig 5:**
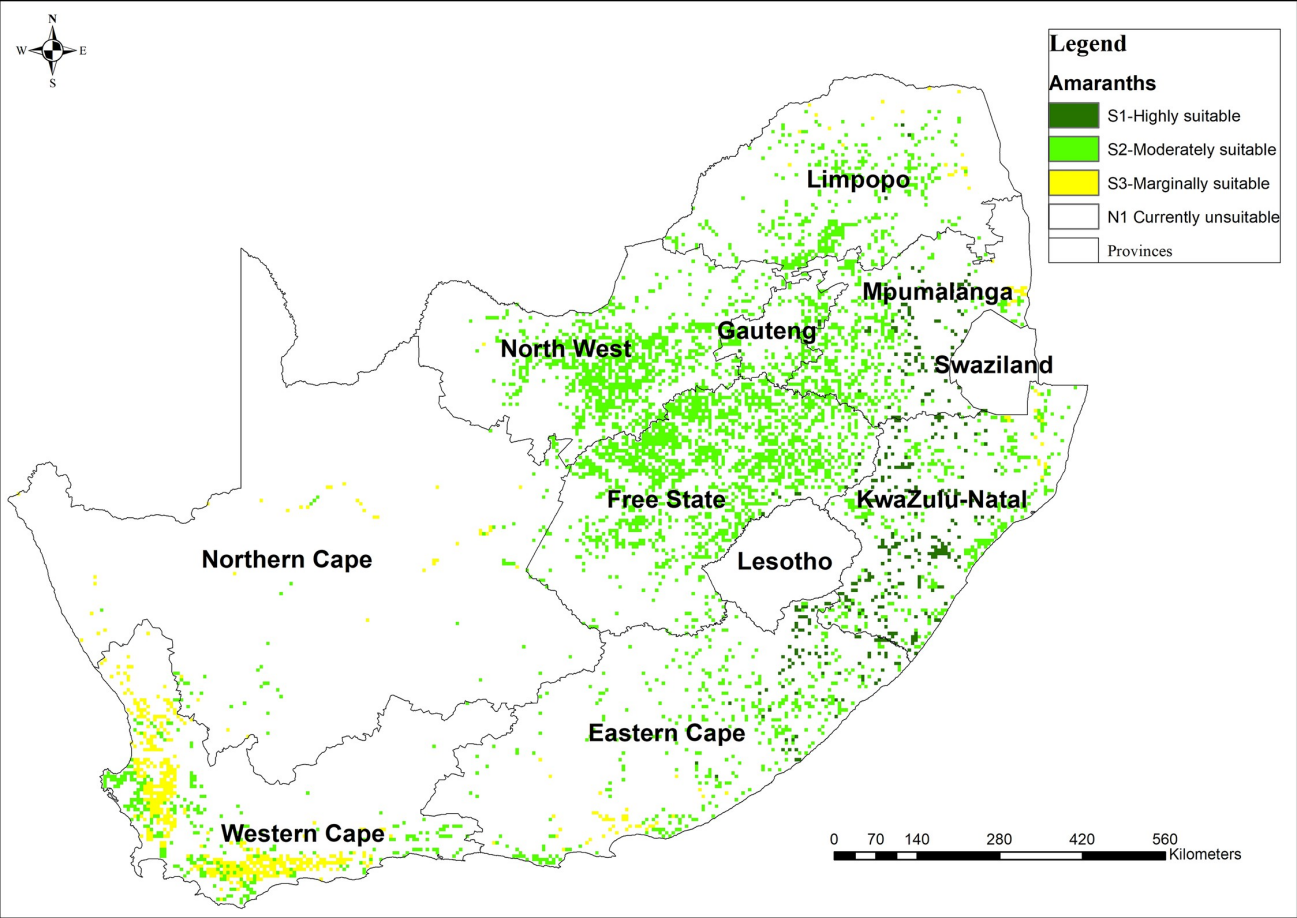
Suitability map for amaranth production in South Africa computed using MCDA-AHP and OWA operators. [Source, *South African Quaternary Catchments database*, *(*https://doi.org/10.6084/m9.figshare.13179881*)*, *in ArcGIS 10*.*5]*.

The land suitability analyses indicated that amaranth is highly suitable across South Africa. The results indicated that there is about 8% of the land that is highly suitable (S1) for the production of amaranth. Moderately suitable (S2) land constitutes the most substantial proportion with 81% of the calculated arable land of South Africa (12 655 859 ha) while marginally suitable (S3) constitutes 11% of calculated arable land (Fig 5). Amaranth is high suitable across South Africa in most cropping areas, even in the Western Cape, where the investigated crops had low suitability (Fig 5). The observed suitability could be associated with the growth requirements of the crops that allow for its production even under marginal conditions. From field visits, farmers confirmed that amaranth is suitable and grow naturally in KwaZulu Natal environments.

### 3.5 Water requirement satisfactory indices for a period of 1981 to 2017

**Fig 6:**
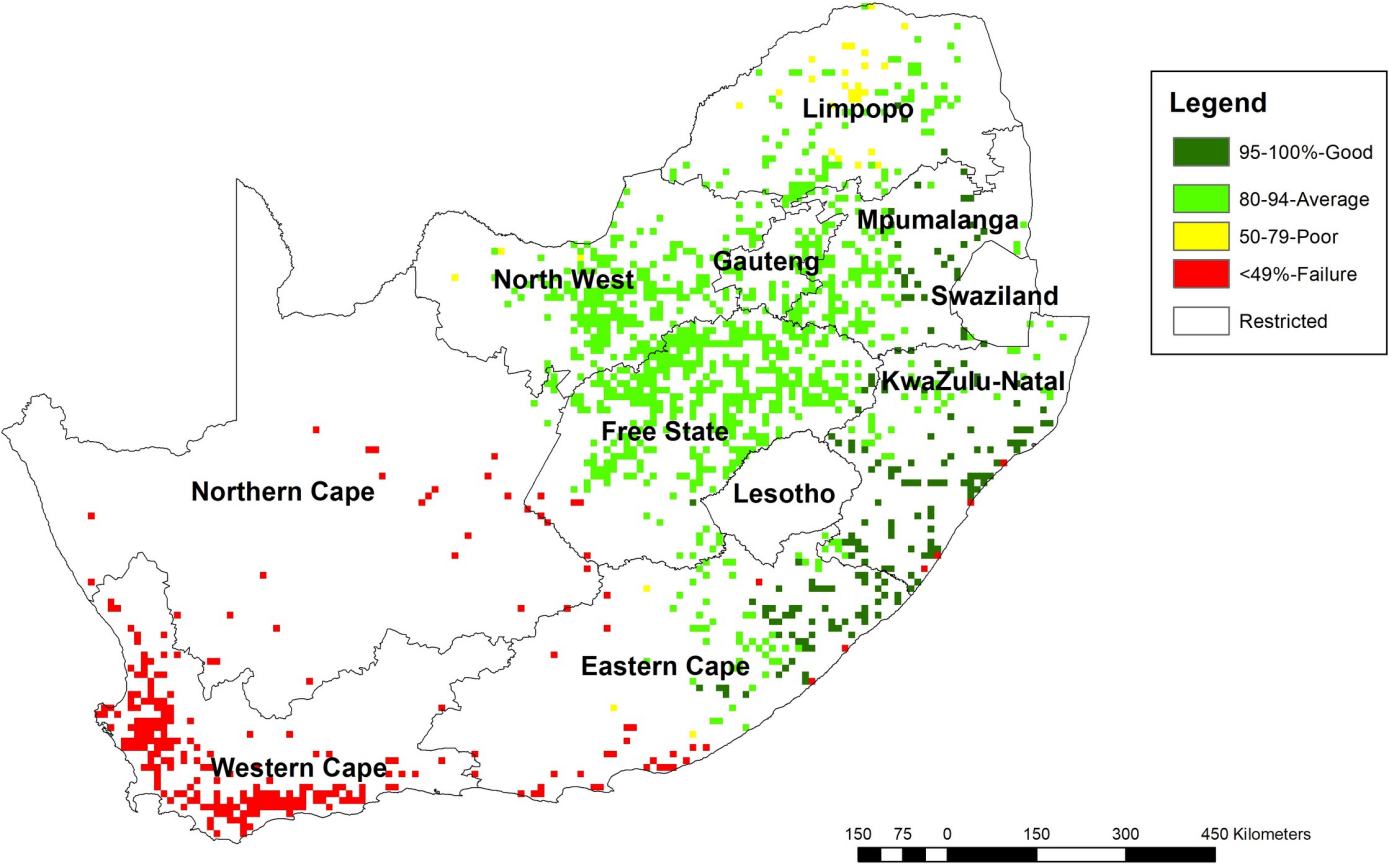
Average water requirement satisfactory indices for a period of 1981 to 2017 in cropping lands in South Africa. [Source, Famine Early Warning Systems Network, (https://earlywarning.usgs.gov/fews, and from USGS Earth Explorer https://earthexplorer.usgs.gov/ and from USGS Earth Explorer https://earthexplorer.usgs.gov/), *in ArcGIS 10*.*5]*.

The Water Requirement Satisfaction Index classification in driest areas of the country, which a mainly the Northern provinces were not applicable. In the Western Cape Province, there was no start of the season, and this is consistent with the low rainfall received and high ET_0_ characteristic of this region Fig 6.

### 3.6 Multi-criteria model accuracy validation

**Fig 7:**
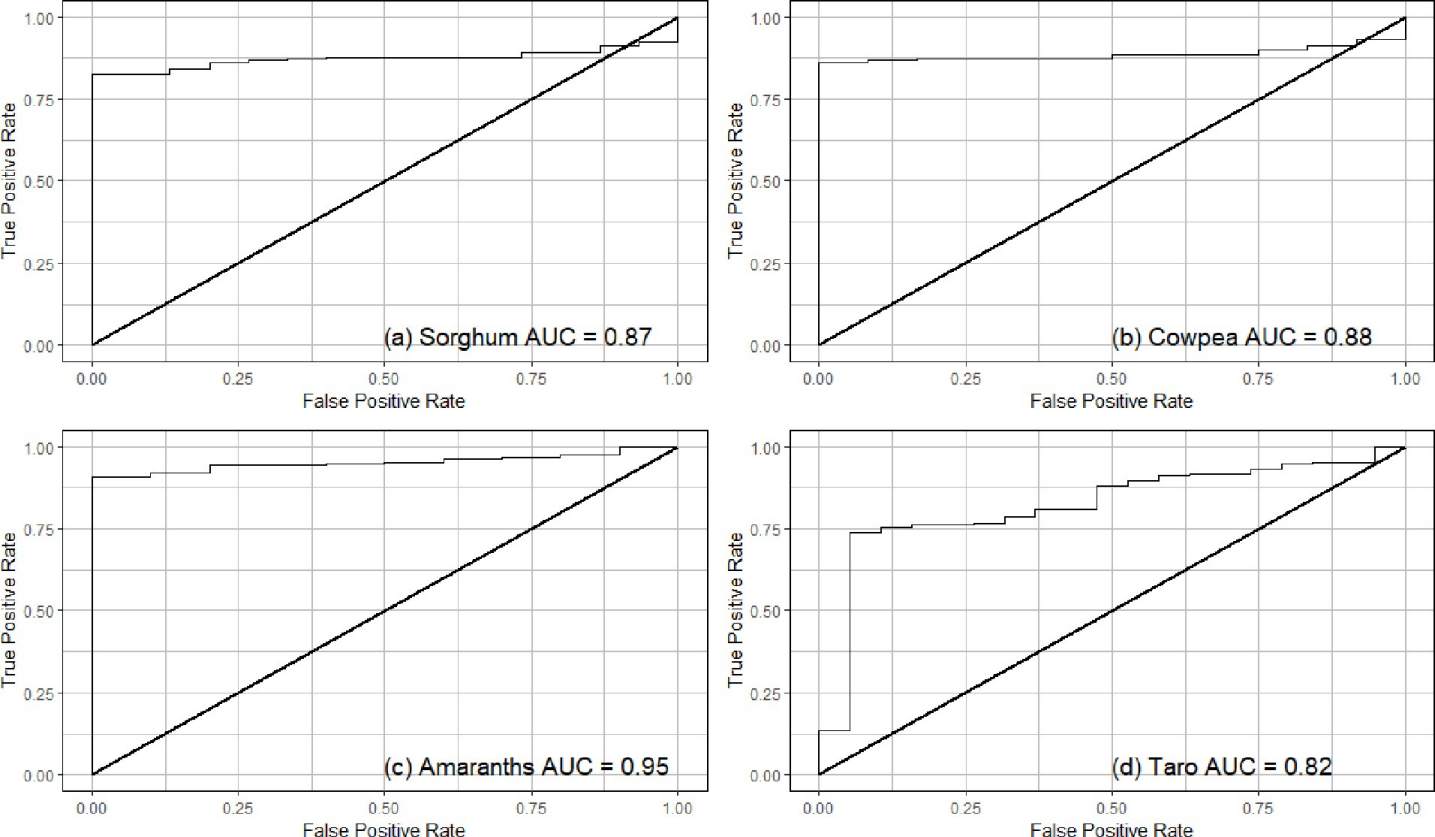
The Receiver Operating Characteristic (ROC), used to generate the Area Under the curve (AUC) which is used for model validation of the logistic regression model for spatial prediction of (a) sorghum, (b) cowpea, (c) amaranth and (d) taro.

The area under curve (AUC) of sorghum (0.87) cowpea (0.88), amaranth (0.95) and taro (0.82) values were greater than 0.5 Fig 7. Considering that the AUCs of all the crops were above 0.8, this indicates that all the models were in this study were accurate in estimating the NUS suitability.

## 4. Discussion

In this study, we assessed the land suitability of NUS using climatic, soil-landscape, as well as socio-economic factors. Use of AHP provides scope for combining expert opinion with measurements in making pairwise comparisons between criteria at each level of the hierarchy to come up with relative weights. According to the local experts’ judgment, rainfall was the most critical variable, followed by temperature, while soil depth and distance from the road were least important (Table 3). The ranking of the variables is somewhat consistent with what was reported as important crop limiting factors for South Africa [75]. Malczewski [35] noted that the relationship between the objectives and attributes has a hierarchical structure. The consistency ratio was calculated as 0.05 (Table 3) and is considered as acceptable [23, 76].

To reduce the risk associated with over-fitting or noise modelling, nine thematic input layers were used by employing matrix pairwise comparison. The matrix pairwise comparison was obtained from different expects, and factor weights were calculated using a pairwise comparison matrix (Table 3). The accuracy of weights used is subjective as it depends on expert opinion; however, the results of the relative weights were used in land suitability evaluation because the Consistency Ratios were within the established acceptable limits (0.1) [76]. The challenge of a deterministic MCDA-AHP method is that assigning weights may be subjective, and the setting of weights represent imprecise point estimates, and the process does not indicate error or confidence [28]. However, the use of AHP methodology provides scope for combining expert opinion with measurements [36, 77, 78]. Expert opinion weighted distance from the road with the lowest weight (Table 3), because the social-economic factor does not affect crop growth directly, but it influences the adoption of NUCS by farmers. Accessibility to markets is highly influenced by road network because it affects markets. There are other socio-economic factors (availability of extension services, access to markets and credit etc.), which can be included in MCDA to develop cropland suitability mapping [79].

Based on the analyses, there are potential environmental benefits to growing NUS in SA. The introduction of NUS into regions classified as moderately suitable (S3) to highly suitable (S1) could increase the crop choices available, and also contribute to biodiversity (SDG 15). The low environmental impacts and increased biodiversity brought about by the introduction of NUS can be viewed as a climate change adaptation strategy (SDG -13) for increasing farmer resilience [80]. More so, for marginalised farming communities that have limited access to improved technologies such as hybrid seed and fertilisers [81]. In this regard, the introduction of NUS into existing cropping systems can be viewed as a sustainable intensification approach [82]. Also, promoting NUS in marginal lands can contribute to food and nutrition security (SDG 2), poverty alleviation (SDG 1) through creating new value chains and human health and wellbeing (SDG 3).

The area under the curve (AUC) of sorghum, cowpea, amaranth and taro was above 0.8 indicating that the land classification based on the logistic regression were highly accurate (Fig 7). These high accuracies could be explained by the robustness, holistic nature and optimal performance of the GIS based MCDA and AHP modelling which was able to characterise land that is optimal for the NUS production in this study. Sun et al. [83], provide an essential guarantee of the AHP model as a decision-support tool for improving the efficiency of water use. Amongst the four crops, taro had the lowest AUC because the crop generally has a high water requirement compared to the other crops [64, 84].

The results of total area suitable for the production of sorghum, taro, and cowpea were consistent with what has been reported to be available arable land (approximately 10.3%) in South Africa [85]. About 70% of South Africa’s land is categorised as unsuitable for rain-fed crop production due to a combination of poor rainfall distribution and soils with low fertility, yet NUS are naturally suitable in marginal areas. However, there were variations in the magnitude of suitability for each of the NUS crops investigated. The results indicated that sorghum and cowpea were suited for drought and heat stress-prone areas such as KwaZulu-Natal, Eastern Cape and Limpopo provinces where the majority of agricultural households reside [31]. Based on AHP analysis, these crop species are, therefore, well adapted to high climate risk and can be produced under water-limited and extremely hot (33–38°C) conditions. Amaranth was highly suitable across most cropping lands in South Africa, and this is because the crop has a short growing period and low water requirement [65].

The suitability of taro in KwaZulu-Natal, Mpumalanga and Gauteng provinces is consistent with the observed length of the growing period, Specifically, taro takes up to 300 days to mature and it has high water use rate (651–1 701 mm) [86]. In this regard, the areas suitable for taro production in South Africa were low and mostly confined to areas receiving high rainfall. The high water requirements would suggest that the crop may be more suited for areas prone to flash flooding as it is also tolerant to aeration stress [86]. Therefore, our results can be used to indicate areas where the investigated crops can be introduced as part of sustainable intensification approaches for climate change adaption [87]. The results are vital in increasing the options for crop choice for marginalised farmers throughout South Africa. However, the information on suitability needs to be complemented with information on "better bet" agronomic management to realise the full potential of the crops in question [9]. Cowpea, sorghum, and amaranths are highly suitable in areas which receive more than 500 mm per season and most of these areas are highly urbanised (i.e. Gauteng province). Therefore, the opportunity cost of promoting NUS near urban areas might be affected by the land value near urban areas, then high valued horticultural crops and dairy production with higher market demands are more preferred by peri-urban farmers [9].

Our methodology focused on assessing crop suitability using mainly physical factors and a single socio-economic factor. Neglected and underutilised crop species are important within smallholder farming systems and address several socio-economic indicators such as widening food value chains, increase food and nutrition security and reducing gender inequality [88]. Promoting or introducing NUS in mapped zones can be an essential part of the solution towards addressing food insecurity, specifically malnutrition, reducing vulnerability to climate variability and change, environmental degradation, and gender inequality. It is argued that holistic land suitability maps, which take into consideration several socio-economic indices, could be more useful to policy-makers and enhancing the participation of marginalised farmers in the food system [6]. The exclusion of key socio-economic indicators in developing suitability maps might affect uptake and adoption of these crop species in areas where they are found to be biophysically suitable. Therefore, to generate information of socio-economic indicators, there is need for future studies to identify innovative ways to derive maximum value from the possible integration of GIS with block-chain, big data, and Internet of Things (IoT) technologies to mine updated data, especially on climatic data and social-economic factors [89, 90]. To achieve this, farmers, private sector and the government will need to support further research on NUS value chains.

The results show that NUS are suitable in a wide range of agro-ecological zones, especially in areas observed to have high ecological risks. Therefore, mainstreaming them into existing systems as alternative crop species to commercially important crops might be a sound adaptation strategy to climate variability and change. However, the interpretation of our results relative to climate change is limited by the fact that we used a historical data set (1950–2000). While this spans across half a decade, most of the extreme climate hazards have been observed in the last 30 years (1990 –present) [91]. As such, future studies should focus on using data from global circulation models (GCMs) to inform climate change scenarios more specifically. However, the current maps remain useful in identifying areas that are currently suitable for NUS production for the first time in South Africa.

High coefficient of determination between MCDA-AHP and WRSI indicated that the climatic parameters used were sufficient to delineate marginal areas within South Africa. Water Requirement Satisfaction Index was developed by the FAO and mostly used by FEWSNET to monitor and investigate crop production in agricultural drought-prone parts of the world [92]. It is used to monitor crop performance during the growing season and based upon how much water is available for the crop by calculating a ratio of actual to potential evapotranspiration [92]. These ratios are crop-specific and are based upon crop development and known relationships between yields and drought stress [92]. Short duration crops such as amaranth and crops that have a low water requirement fit well in all environments of South Africa. While the WRSI uses climate-related stress factors other than soil available water, the relationship between two independent classifications showed that this study’s NUS land suitability was satisfactory. The negative coefficient of determination (R = -0.15) observed for taro suitability, and WRSI might be due to crop water requirements and length of the growth period, which overlaps into the dry season.

Taro is predominantly a wetland crop; however, upland varieties exist and these have been shown to have lower levels of water use and also to possess drought tolerance through avoidance and escape mechanisms [86]. However, escape mechanisms (i.e. phenological plasticity) make taro suitability to be negatively correlated with WRSI. One of the significant limitations of WRSI index is that it uses satellite-based rainfall estimates which are influenced by cold-cloud-duration (CCD) especially in February to March because of overcasting clouds in subtropics. Therefore, there is a degree of error that could influence WRSI classification, especially on the balance of evapotranspiration in a lean season in South Africa [93, 94]. To overcome these challenges, future studies could employ unarmed aerial vehicles derived data with very-high-spatial resolution images and LiDAR (Light Detection and Ranging) technology, which can provide 3D models of farmland [95]. LiDAR technology could provide accurate maps of natural resources and farmlands for sustainable production of NUS in South Africa [96, 97].

Sorghum, cowpea and amaranth have characteristics that allow them to grow under water-stressed environments compared to major crops which is in agreement with WRSI classification [92]. This means selected NUS could make use of land that is unsuitable for growing cash crops, offering a complement crop production scenario rather than a substitution production scenario (Mabhaudhi et al., 2019). This study is a first step towards the reclassification of land in South Africa in acknowledgement of NUS in national cropping systems.

## 5. Recommendations

The land suitability maps generated in this study can be used to indicate where NUS can be promoted as alternative crop choices or to complement the current range of crops grown within marginalised cropping systems. As such, the maps can be used to inform site-specific crop diversification recommendations as a sustainable intensification strategy [87]. To mainstream NUS into cropping systems found in the delineated regions of suitability maps developed in this study, a transdisciplinary approach is required. Moreover, there is a need to create a conducive environment for all participating stakeholders. This can be achieved if there is a harmonisation of existing policies that speak to land, environment, agriculture and health, and new policies on land use are co-designed based on evidence. Policies such as the National Environmental Management: Biodiversity Act of 2004, National Food and Nutrition Security Policy [98] and Draft Policy on Preservation and development of Agricultural Land Bill 2015 could foster co-development of NUS technologies and aid in addressing challenges in the land, environment, agriculture and health domains;

We identified several challenges in defining the suitability of NUS. Key among these included urbanisation and increase in food and nutrition insecurity, bush encroachment, as well as the competition between agriculture and protected natural habitats. In this regard, agronomists, climatologists, ecologists and economists need to collaborate in co-designing the suitability indices to inform policy and practice. Such collaborations will ensure that suitability maps for NUS are holistic and relevant in addressing crosscutting challenges. To make current land suitability maps more relevant to addressing global grand challenges, researchers need to consider the inclusion of socio-economic parameters. The AHP is one of the most relied on methods in MCDM; however, the consistency is difficult to achieve where there are more than nine criteria/indicators under consideration [23]. Nevertheless, its ability to measure consistency is one of the factors that gives the it an edge over other methods. Therefore, parameters considered in MDCM should be context-specific and informed by an outcomes-based approach.

While our results remain applicable for use, future research should consider using data with a finer resolution to improve the accuracy of mapping. This will aid in improving delineation of land suitability in marginalised agricultural communities that are known to be highly heterogeneous. The application of unarmed aerial vehicles could be used to validate satellite-derived data and to capture high-resolution images. One such sensor is LiDAR (Light Detection and Ranging) technology, which can provide 3D models of farmland [95]. LiDAR technology can provide accurate maps of natural resources and farmlands for sustainable production of NUS in South Africa [96, 97]. The use of high-resolution images in developing land suitability of NUS is of utmost importance in solving land use challenges. However, the process is often difficult, labour intensive and costly. The return on investment (ROI) of using LiDAR in delineating areas suitable for NUS may be low as NUS still lack developed markets and value chains [99]. Overall, the cost benefit of using LiDAR for smallholder farmer settings needs to be evaluated to determine the feasibility of such investments [99].

Climate change is projected to shift current agro-ecological zones and land-use patterns [86]. We recommend that land suitability analysis should include climate scenarios in their simulation. The inclusion of climate scenarios in land suitability analysis will allow for more proactive agricultural planning by informing policies such as the National Climate Change and Health Adaptation Plan on projected suitability of agricultural land to produce diverse crops in the short-, medium- and long-term.

Future studies should focus on using new predictive tools in forecasting. It is observed that the majority of the studies in resource allocation utilised primitive GIS techniques. The future studies should focus on combining the Environmental Policy Integrated Climate (EPIC) models with other methods for assessing the spatial distribution and stimulating the production of crops. The EPIC model is used for predicting crop production levels incorporating the near-real-time changes in crop environment can be integrated with other techniques for improved decision making.

## 6. Conclusion

We investigated the potential spatial suitability distribution for sorghum, cowpea, amaranth and taro in South Africa. This study used AHP model in GIS to integrate nine multidisciplinary thematic factors from climatic indicators from 1950 to 2000 (seasonal rainfall, seasonal maximum and minimum temperature), soil and landscape attributes (soil depth, slope, elevation), social-economic (road) and technical indicators (LULC). Rainfall was the most critical variable and criteria with the highest impact on land suitability of the NUS in this study. Neglected and underutilised crop species have the potential to be grown on marginal land, and they can complement major crops and create greater diversity in cropping systems for building resilient cropping systems. The analysis indicated that sorghum, cowpea and amaranth have the potential to be grown in marginal areas in S3 zones where land has moderate limitations for agricultural use. The suitability for sorghum, cowpea, and amaranth concurred with the water requirement satisfactory index (WRSI). Matching crop requirements with available resources through land suitability analysis is essential to sustainable agriculture.

Mapping NUS production potential zones in SA is key to promoting NUS production by providing evidence to assist decision- and policy-makers on crop choice. Specifically, the results are useful in informing the Climate Smart Agriculture Strategy, National Policy on Comprehensive Producer Development Support and Indigenous Food Crops Strategy that are currently under development in South Africa. The suitability maps are also useful for informing decisions on climate change adaptation (climate-smart agriculture) and sustainable agriculture practices as well as informing decisions on the creation of markets for NUS.

The findings are useful in informing land-use classification, especially in marginal environments. The method used can be adopted to other SSA countries and other regions that share a similar context with regards to promoting cultivation of NUS. Promoting NUS within marginal production areas has the potential to create new and sustainable economic pathways and improve availability and access to nutrient-dense foods. The importance of smallholder farmers to sustainable food systems, and their participation in local food systems, must be emphasised. Finally, policies such as the National Food and Nutrition Security Policy and National Developmental Plan of South Africa [100] need to give a clear road map for NUS production, especially by explicitly mentioning NUS and targeting them for production on marginal lands that are currently not suitable commercial crops production as a strategy to improve food and nutrition security within these areas.

## Supporting information

S1 Fig Spatial distribution of seasonal precipitation, for period of 1950–2000 for South Africa, [Source, *South African Quaternary Catchments database*, *(*https://doi.org/10.6084/m9.figshare.13179881*)*, *in ArcGIS 10*.*5])*, *in ArcGIS 10*.*5]*. (TIF)

S2 Fig Seasonal average maximum temperature for South Africa for period of 1950–2000, [Source, *South African Quaternary Catchments database*, *(*https://doi.org/10.6084/m9.figshare.13179881*)*, *in ArcGIS 10*.*5])*, *in ArcGIS 10*.*5]*. (TIF)

S3 Fig Seasonal average maximum temperature for South Africa for period of 1950–2000, [Source, *South African Quaternary Catchments database*, *(*https://doi.org/10.6084/m9.figshare.13179881*)*, *in ArcGIS 10*.*5])*, *in ArcGIS 10*.*5]*. (TIF)

S4 Fig Reference crop evapotranspiration (ETo) millimetres (mm) for South Africa, [Source, *South African Quaternary Catchments database*, *(*https://doi.org/10.6084/m9.figshare.13179881*)*, *in ArcGIS 10*.*5]*. (TIF)

S5 Fig Length of growing period (LGP) for South Africa, [Source, *South African Quaternary Catchments database*, *(*https://doi.org/10.6084/m9.figshare.13179881*)*, *in ArcGIS 10*.*5]*. (TIF)

S6 Fig Soil depth suitability map for South Africa, [Source, *South African Quaternary Catchments database*, *(*https://doi.org/10.6084/m9.figshare.13179881*)*, *in ArcGIS 10*.*5]*. (TIF)

S7 Fig Elevation suitability map for South Africa, [Source, *South African Quaternary Catchments database*, *(*http://earthexplorer.usgs.gov*)*, *in ArcGIS 10*.*5]*. (TIF)

S8 Fig Crop slope suitability for South Africa, [Source, *South African Quaternary Catchments database*, *(*https://doi.org/10.6084/m9.figshare.13179881*)*, *in ArcGIS 10*.*5*. (TIF)

S9 Fig Crop production suitability map for land use land cover map for South Africa. [Source, *South African Quaternary Catchments database*, (https://figshare.com/s/2a7d1d5c37a6674196c3), *in ArcGIS 10*.*5]*.
(TIF)

S10 Fig Distance from road suitability map for South Africa, [Source, *South African Quaternary Catchments database*, (https://figshare.com/s/7668a4c641a0ff3d0c03), *in ArcGIS 10*.*5]*. (TIF)

S1 Appendix (DOCX)

S1 File (R)
