# Correction: Multi-criteria suitability analysis for neglected and underutilised crop species in South Africa

**DOI:** 10.1371/journal.pone.0259427

**Published:** 2021-10-27

**Authors:** Hillary Mugiyo, Vimbayi G. P. Chimonyo, Mbulisi Sibanda, Richard Kunz, Luxon Nhamo, Cecelia R. Masemola, Carole Dalin, Albert T. Modi, Tafadzwa Mabhaudhi

There is an error in the seventh author’s name. The correct name is: Carole Dalin. Author Carole Dalin’s ORCID iD is: 0000-0002-2123-9622 (https://orcid.org/0000-0002-2123-9622).
