# Supplementary figures and images for: Multi-criteria suitability analysis for neglected and underutilised crop species in South Africa
Source: PLoS One. 2021 Jan 19;16(1):e0244734. doi: 10.1371/journal.pone.0244734 (PMC7815157; doi:10.1371/journal.pone.0244734)

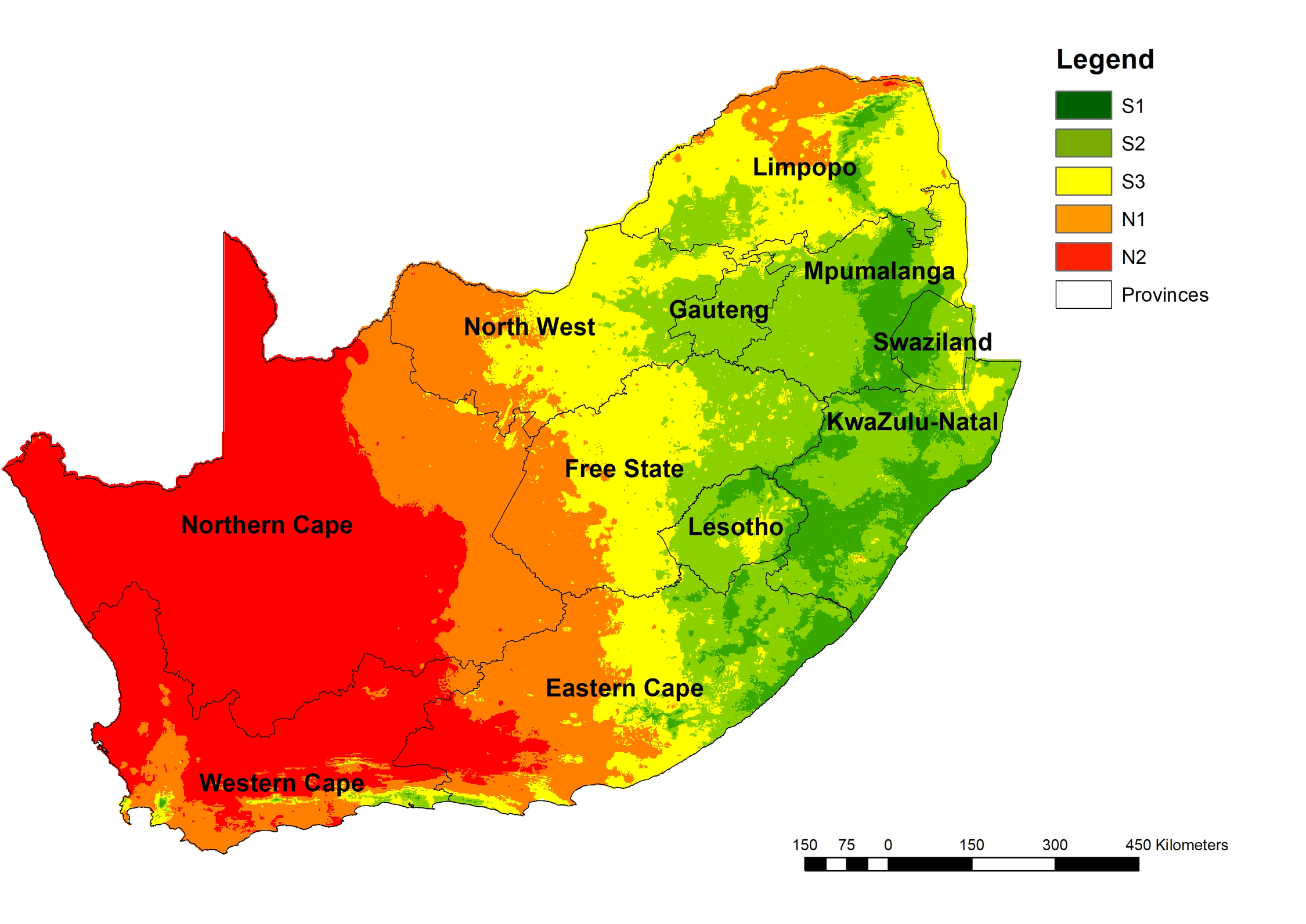

Supplement: S1 Fig — (TIF) [file pone.0244734.s001.tif]

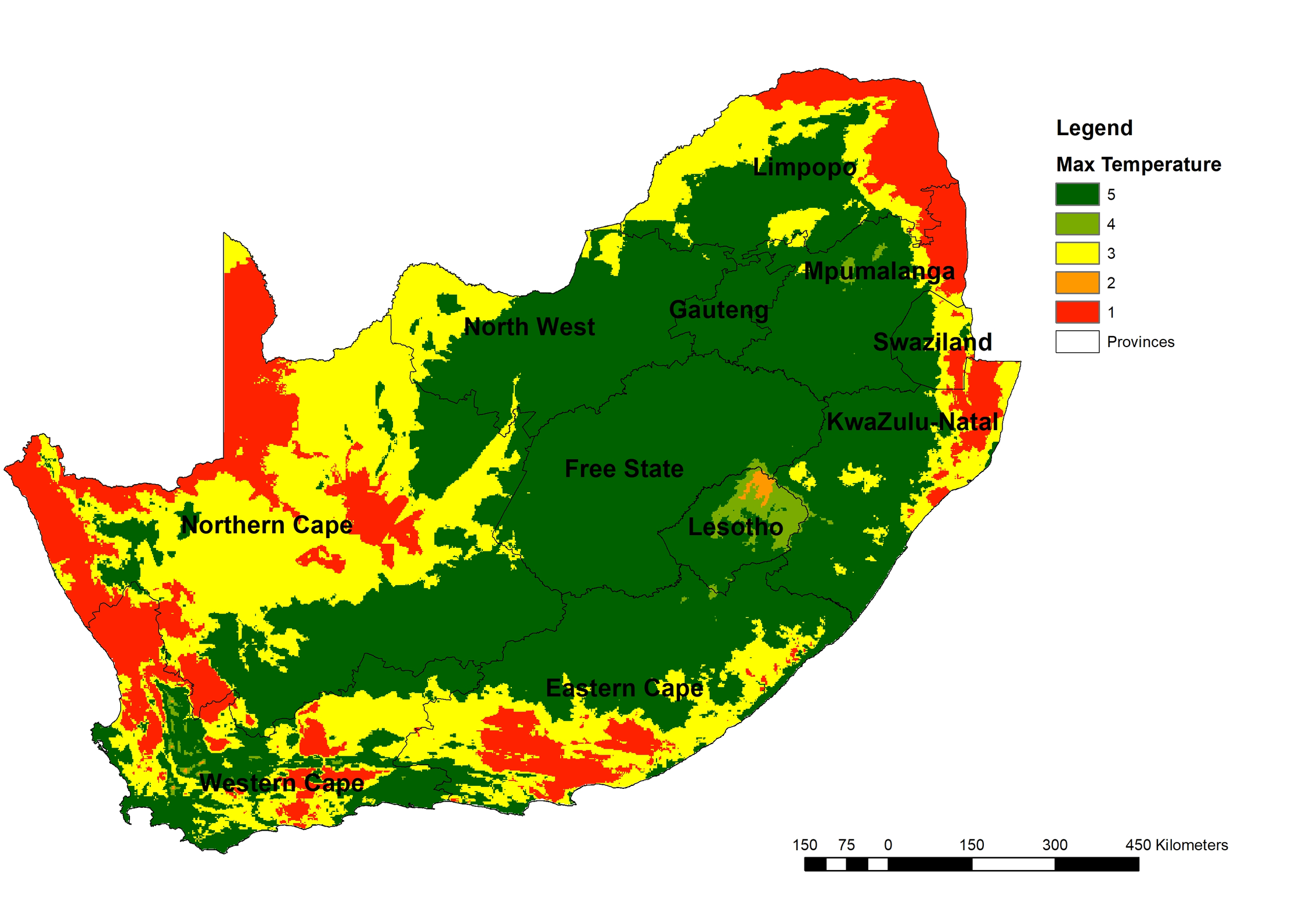

Supplement: S2 Fig — (TIF) [file pone.0244734.s002.tif]

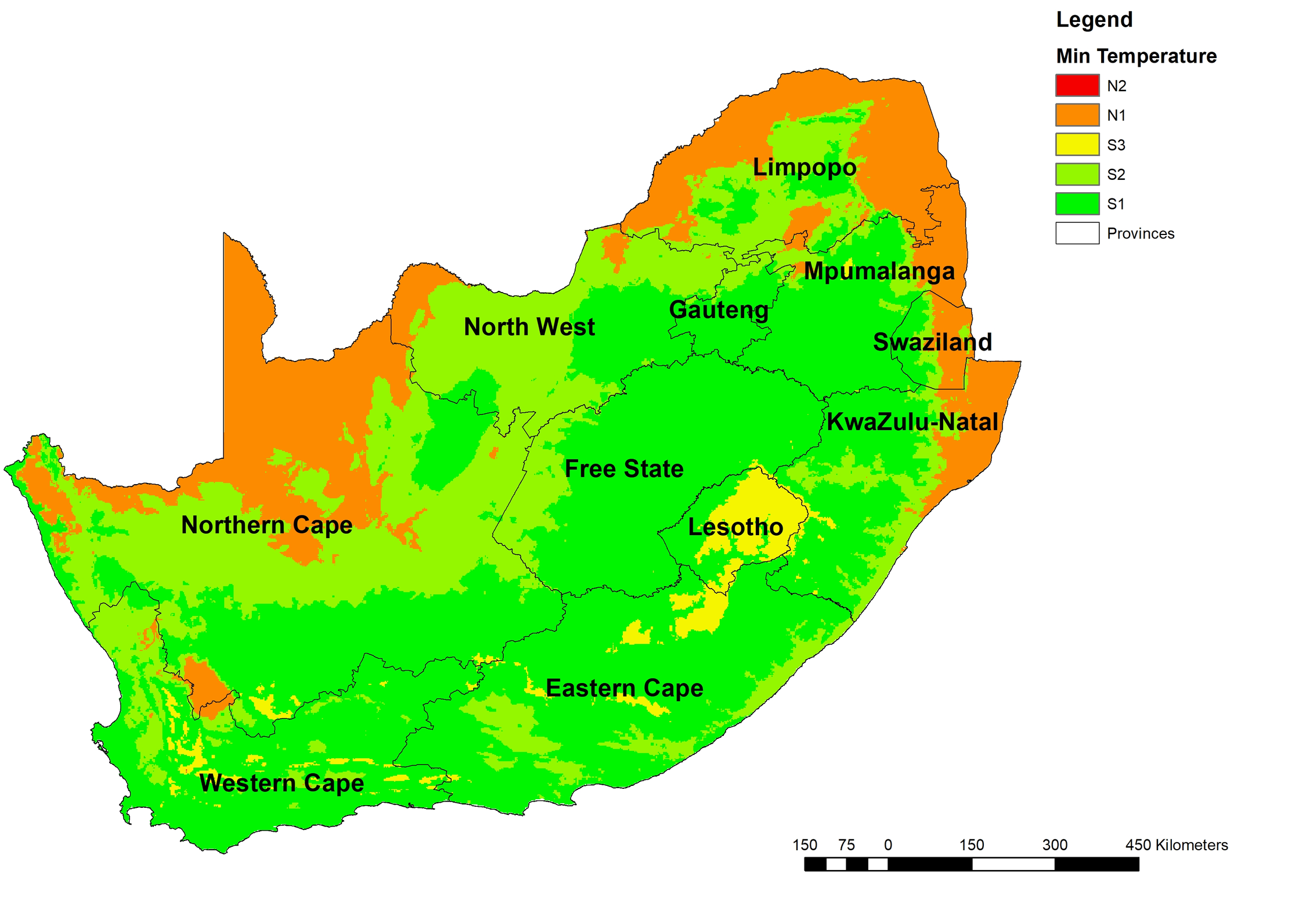

Supplement: S3 Fig — (TIF) [file pone.0244734.s003.tif]

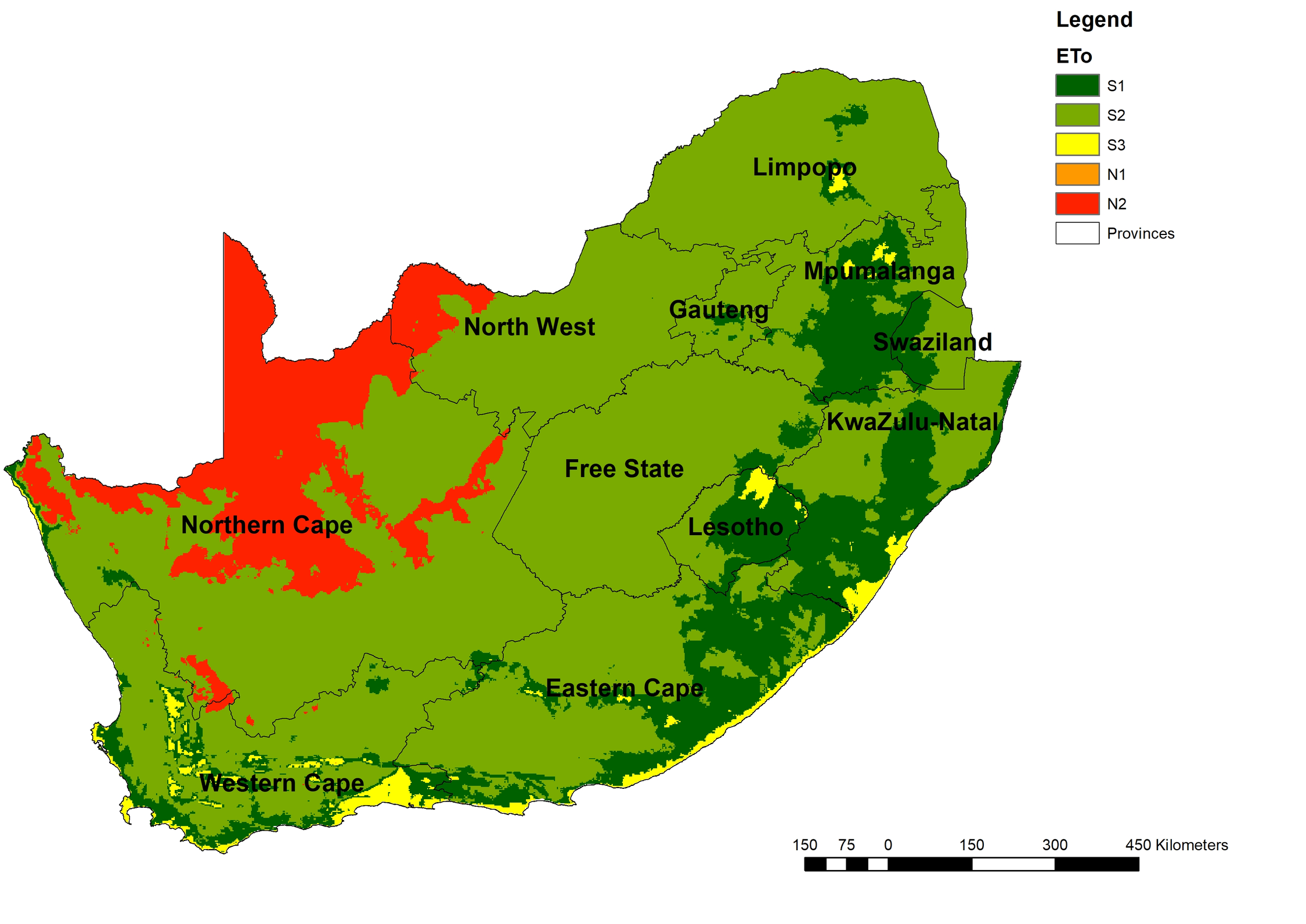

Supplement: S4 Fig — (TIF) [file pone.0244734.s004.tif]

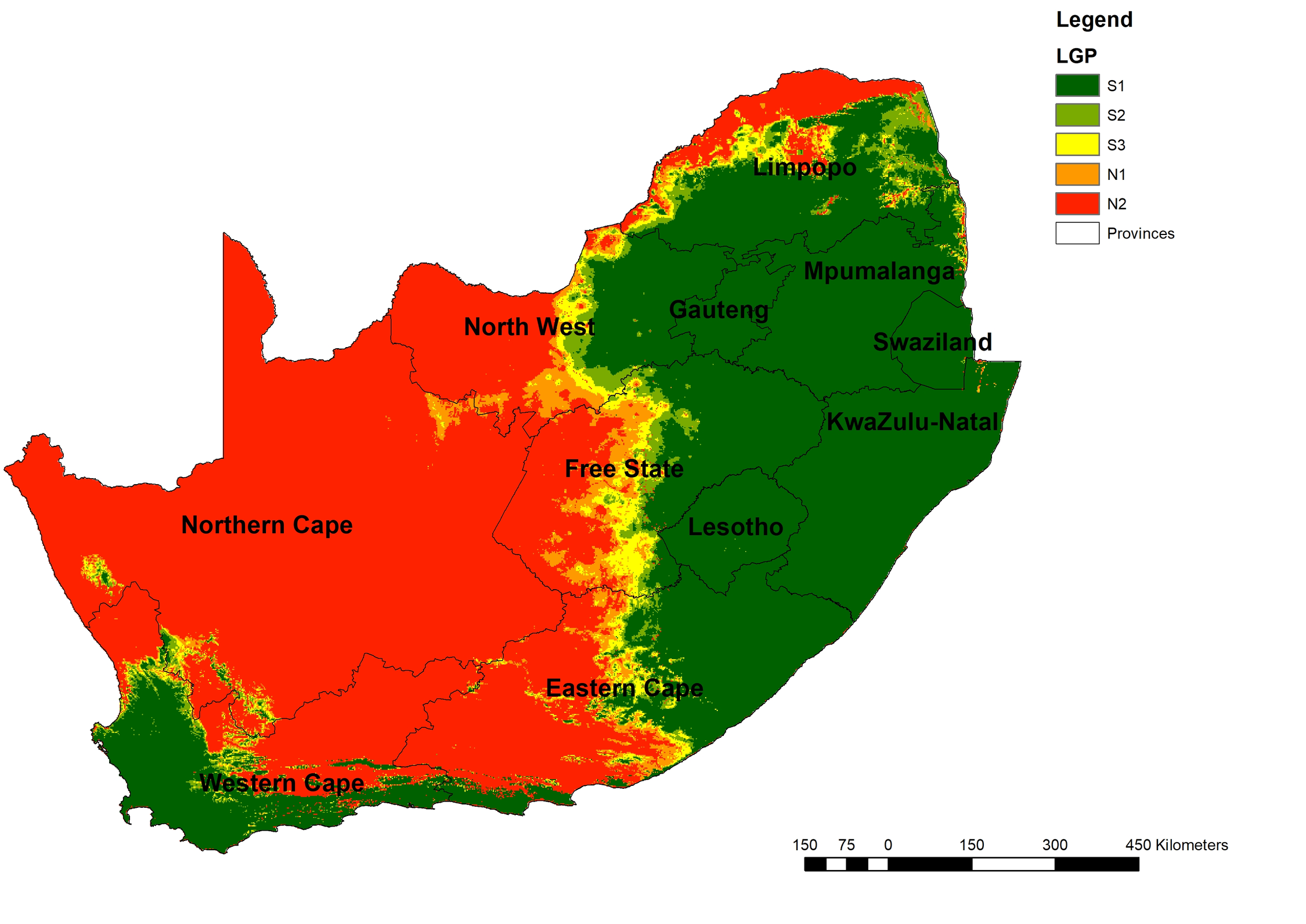

Supplement: S5 Fig — (TIF) [file pone.0244734.s005.tif]

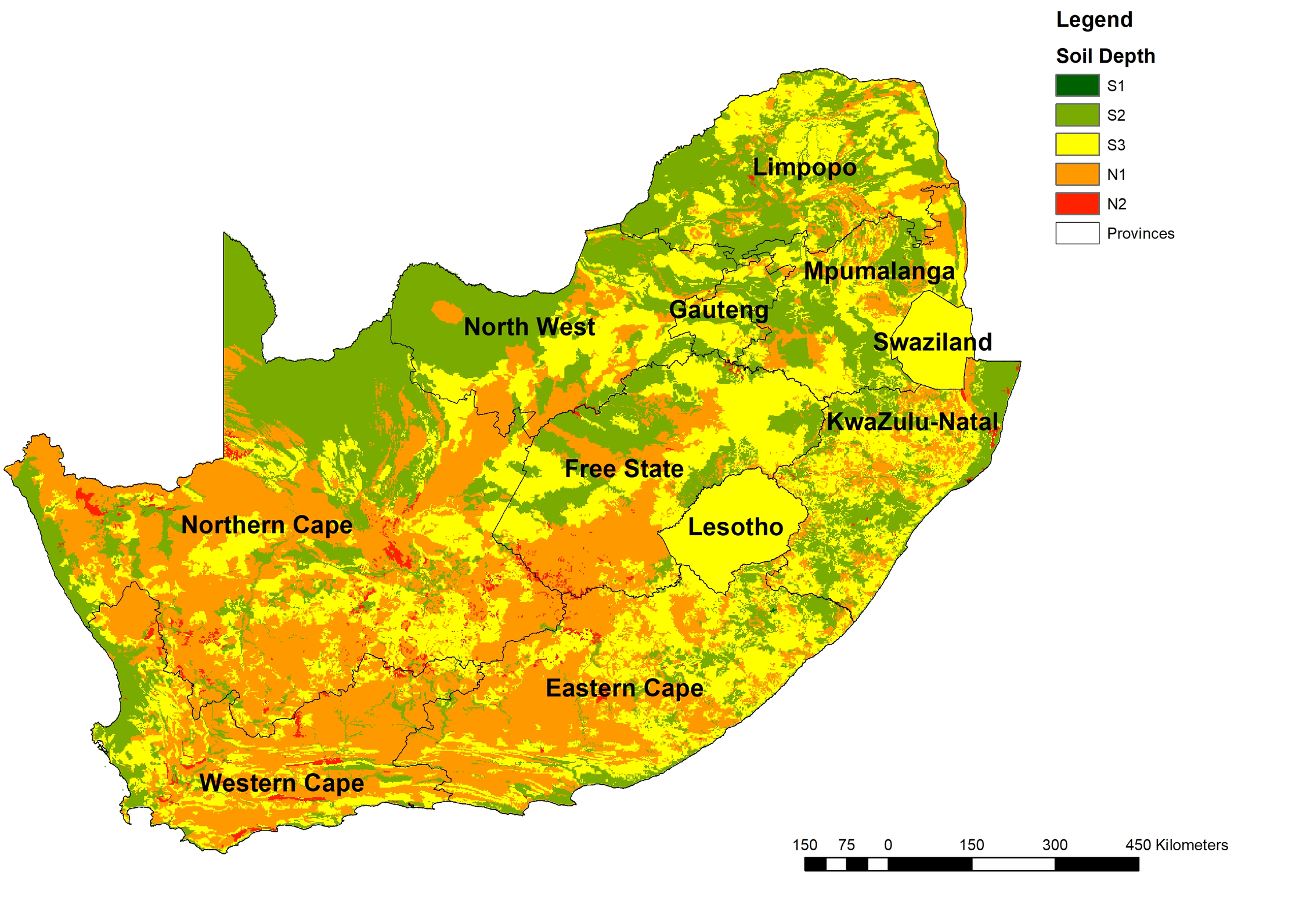

Supplement: S6 Fig — (TIF) [file pone.0244734.s006.tif]

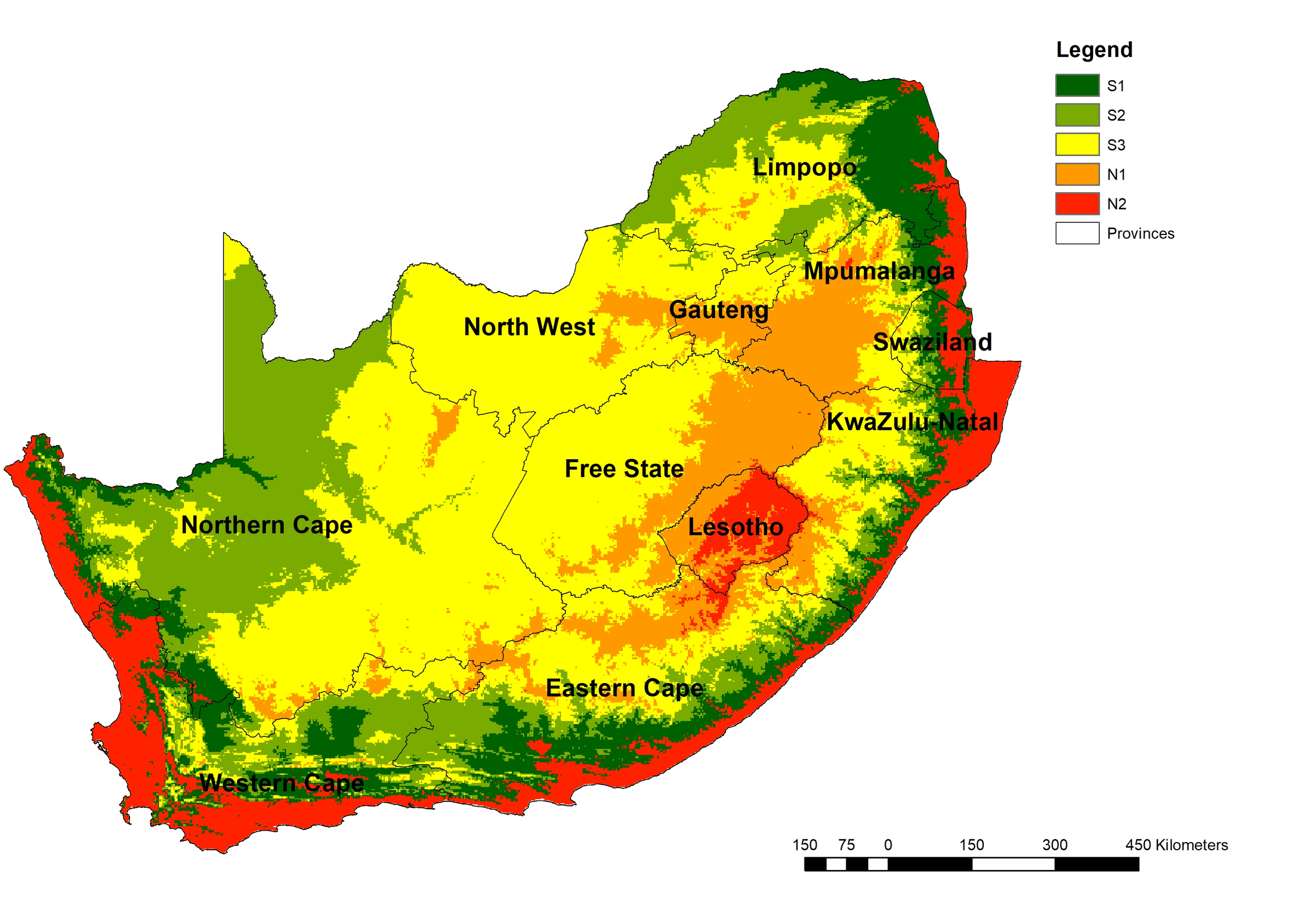

Supplement: S7 Fig — (TIF) [file pone.0244734.s007.tif]

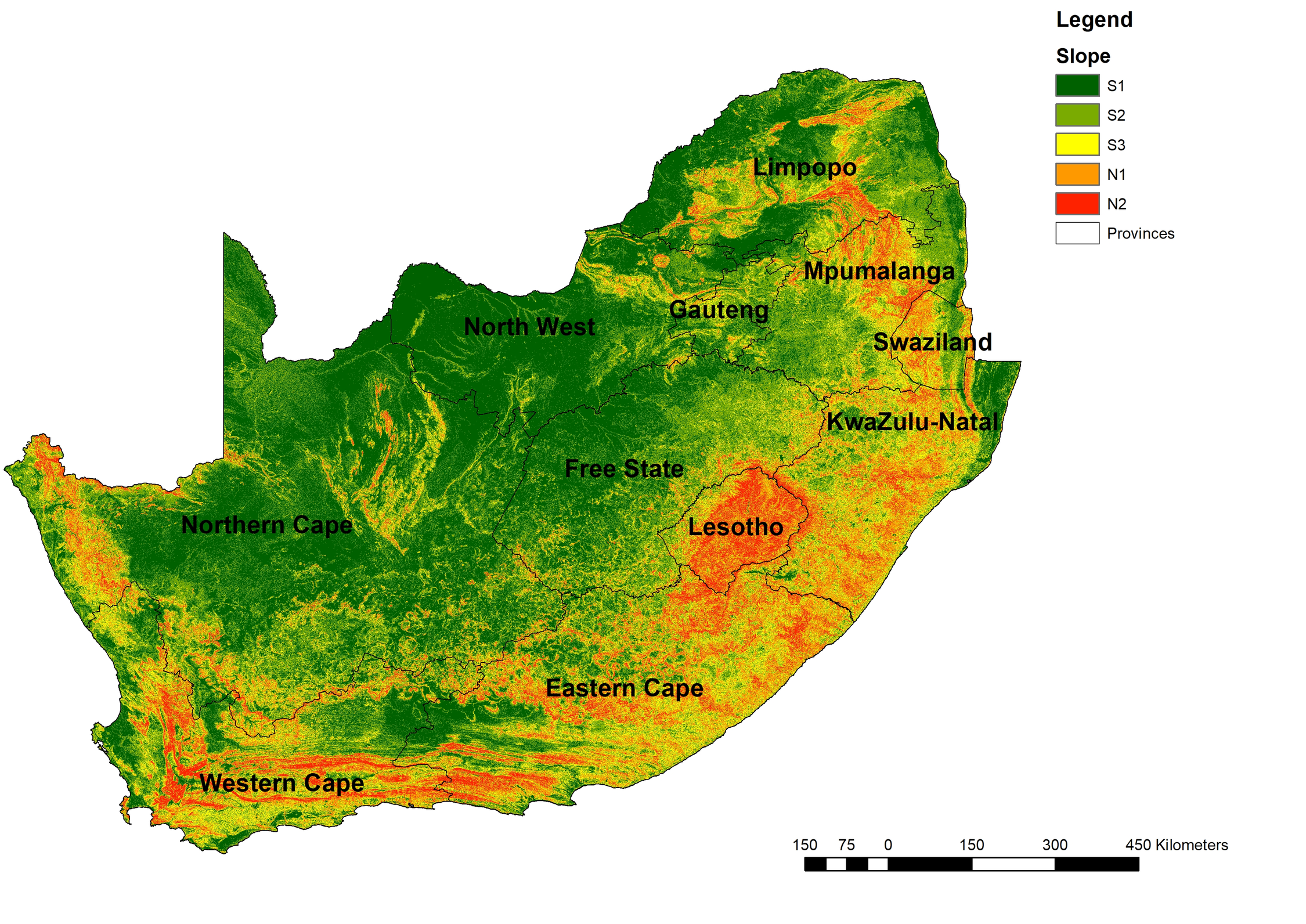

Supplement: S8 Fig — (TIF) [file pone.0244734.s008.tif]

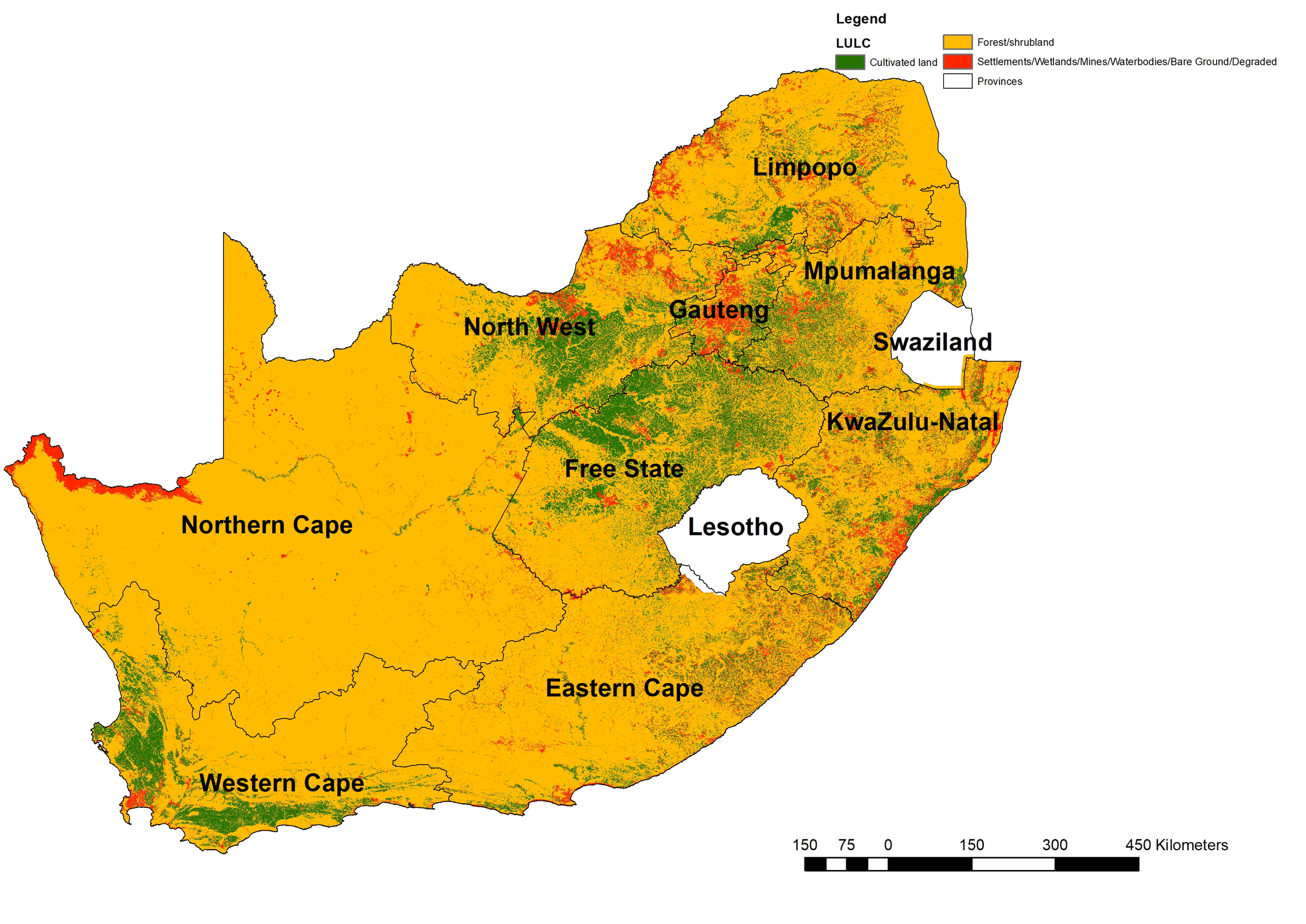

Supplement: S9 Fig — [Source, South African Quaternary Catchments database, (https://figshare.com/s/2a7d1d5c37a6674196c3), in ArcGIS 10.5]. (TIF) [file pone.0244734.s009.tif]

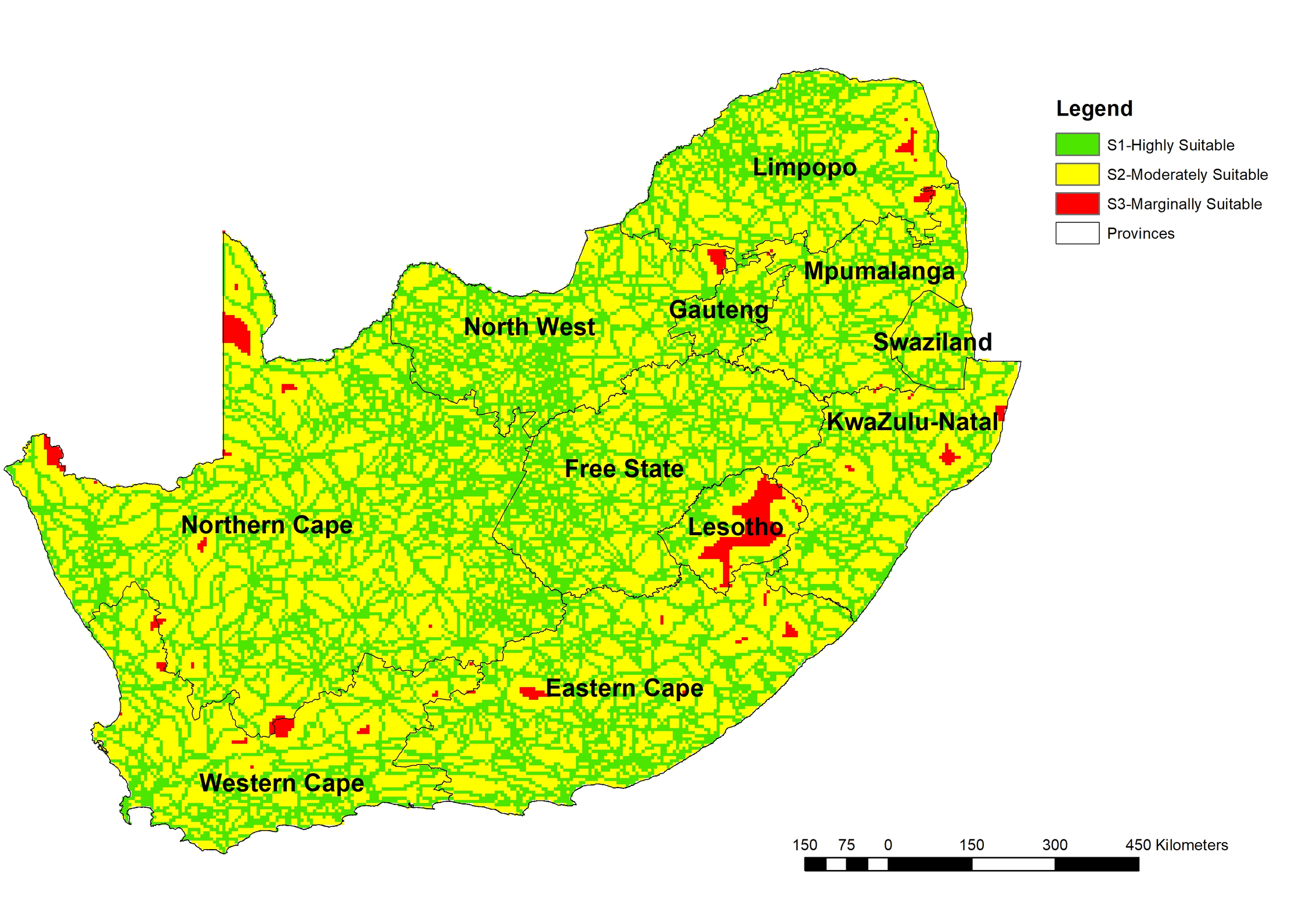

Supplement: S10 Fig — (TIF) [file pone.0244734.s010.tif]
