# Supplementary material for: Multi-criteria suitability analysis for neglected and underutilised crop species in South Africa
Source: PLoS One. 2021 Jan 19;16(1):e0244734. doi: 10.1371/journal.pone.0244734 (PMC7815157; doi:10.1371/journal.pone.0244734)
Supplement: S1 Appendix — (DOCX) [file pone.0244734.s011.docx]

# **Supplementary information**

## The Geography of South Africa

Rainfall is undoubtedly the dominating factor determining crop production, especially in marginal areas where irrigation facilities are limited for smallholder farmers (1). The country is characterized by a mild, temperate climate (2). Precipitation varies spatially across the country with an average annual of 450 mm (compared to a global average of 860 mm), and it is variable across the seasons (3). About 890 mm of precipitation falls yearly in the Eastern Low-veld and the Eastern Uplands as far west as the Drakensberg (3) (S1 Fig ). The High Veld receives about 380 to 760 mm of precipitation annually, the amount diminishing rapidly toward the west (S1 Fig). About 61% of the country receives rainfall of less than 500 mm rainfall annually, which is considered the minimum for successful dryland farming. Where rainfall exceeds 500 mm, major crops include maize, soybean, tobacco, sugar cane and high-value horticultural crops.

South Africa is characterised by a range of thermal zones and length of rain-fed growing days, which both affect the suitability of crops. A different range of soil depth also characterises the country, the highest percentage of medium-sized soil texture and has the highest mountain range of approximately 3482 m in the east of the country (2). A number South African smallholder farmers who have few financial resources, limited access to infrastructure and disparate access to information are located in marginal areas (4). Therefore, smallholder farmers need a transformational adaptation (TA) of agricultural systems to climate change and one of TA strategy is to grow NUS on recommended land units in South Africa.

**Single factor suitability maps**

Table 1: Climatic factors used to delineate land suitability maps for neglected and underutilised crop species

| **Factor** | **Description** | | | **Suitability maps** |
| --- | --- | --- | --- | --- |
| Precipitation (mm) 1.7 km resolution | Precipitation is defined as liquid or solid products of the condensation of water vapour falling from clouds or deposited from the air on the ground (5). Wet periods can be calculated from daily precipitation events like the start of the season, dry spells, end of the season. In SA, precipitation is undoubtedly the dominating factor determining crop production, especially in marginal areas where irrigation facilities are limited for smallholder farmers (Tibesigwa et al., 2017). Precipitation varies spatially across the country with an average annual of 450 mm (compared to a global average of 860 mm), and it is variable across the seasons (Smithers and Schulze, 2000) (Fig 8). | | | S1 Fig.tif, Spatial distribution of seasonal precipitation, for period of 1950-2000 for South Africa, [Source, *South African Quaternary Catchments database,(* <https://doi.org/10.6084/m9.figshare.13179881>*), in ArcGIS 10.5]), in ArcGIS 10.5]* |
| Temperature 1.7 km resolution | Temperature is a measure of heat accumulation and is instrumental in crop growth and management. Temperature is presented as maximum and minimum of air near the earth's surface; the surface of the ground, the soil at various depths. The optimum temperature for photosynthesis is (25°C), and plants growing in a CO_2_ enriched environment thrive in slightly warmer conditions (28°C) (6). The photosynthesis rates drop off sharply if temperatures rise above 30 °C, and it also falls if temperatures are cooler (6,7) (S2 and S3 Fig) | | | S2 Fig. tif, Seasonal average maximum temperature for South Africa for period of 1950-2000, [Source, *South African Quaternary Catchments database,(* <https://doi.org/10.6084/m9.figshare.13179881>*), in ArcGIS 10.5]), in ArcGIS 10.5]*  S3 Fig.tif, Seasonal average maximum temperature for South Africa for period of 1950-2000, [Source, *South African Quaternary Catchments database,(* <https://doi.org/10.6084/m9.figshare.13179881>*), in ArcGIS 10.5]), in ArcGIS 10.5]* |
| Reference crop evapotranspiration (ETo) millimetres (mm) or (lm^-2^)) 1.7 km resolution | Reference crop evapotranspiration refers to evapotranspiration rate from a reference surface, not short of water (8). The reference surface is a hypothetical grass reference crop with specific characteristics (9). Climate factors such as rainfall and potential evapotranspiration are among the crucial factors that affect the suitability of an area for irrigation (10) (S4 Fig). | | | S4 Fig.tif, Reference crop evapotranspiration (ETo) millimetres (mm) for South Africa, [Source, *South African Quaternary Catchments database,(* <https://doi.org/10.6084/m9.figshare.13179881>*), in ArcGIS 10.5]* |
| Length of growing period (LGP) 1.7 km resolution | This represents the number of days when soil moisture and temperature permit crop growth (S5 Fig). The Adapted FAO Approach was used to determine moisture growing season; it assumed that during the period when $P \geq0.3Er$ sustained plant growth can take place, where P is median monthly precipitation (mm) and mean monthly Epan is considered as the reference potential evaporation- Er (11). The growing period was calculated using the moisture growing season by applying a simple water budgeting approach(11,12). | | | S5 Fig.tif, Length of growing period (LGP) for South Africa, [Source, *South African Quaternary Catchments database,(* <https://doi.org/10.6084/m9.figshare.13179881>*), in ArcGIS 10.5]* |
| Water Requirement Satisfaction Index (WRSI)-at 1.0-degree resolution | An indicator of crop performance based on the availability of water during a growing season. Important in locations where weather stations or other ground observations are sparse or non-existent. The indices can be calculated using seasonal actual crop evapotranspiration (AETc) to the seasonal crop water requirement, which is the same as the potential crop evapotranspiration (PETc) (13,14). PETc is crop-specific potential evapotranspiration after an adjustment is made to the reference crop potential evapotranspiration (PET) by the use of appropriate crop coefficients (Kc) (15). Crop coefficients values define the water use pattern of a crop. | | |  |
| Soil and landscape attributes used to delineate land suitability maps for neglected and underutilised crop species | | | | |
| Factors | | Description | Source | |
| Soil depth at 250m resolution | | It is the depth of soil to which the roots of a plant can easily penetrate to withdraw water and extract nutrients from the root zone (Bello and Walker, 2017). Soil depth is the most critical soil property affecting the hydrologic properties of soil and its behaviours against erosion. Water-storing capacity and effective rooting depth are related to soil depth. Effective rooting depth is sometimes related to soil depth, and it is most critical, but unavailability of data at spatial was a challenge. Shallow soils may restrict the development of plant root due to which the plant may suffer adverse conditions in the limited soil volume. Soil depth suitability map is shown in ( S6 Fig) | | S6 Fig.tif, Soil depth suitability map for South Africa, [Source, *South African Quaternary Catchments database,(* <https://doi.org/10.6084/m9.figshare.13179881>*), in ArcGIS 10.5]* |
| Elevation (mm) 30m resolution | | The height of an object above a given level or implied place, especially above sea level (Mendelsohn, 2008). Variation in elevation has an impact on the number of agro-climatic factors like soils, microclimatic effects, and other processes that could affect land suitability (Abera et al., 2018). Elevation affects cropland suitability because of temperature change with an increase of height in the lower troposphere of the atmosphere. The vegetation and vernalisation periods are delayed by 4-6 days for every additional 100 m in elevation on the mountains. In this study, the 30m spatial resolution DEM data of SRTM was acquired from USGS (S7 Fig). | | S7 Fig.tif, Elevation suitability map for South Africa, [Source, *South African Quaternary Catchments database,(* <http://earthexplorer.usgs.gov>*), in ArcGIS 10.5]* |
| Slope | | The slope is a crucial factor affecting vegetation structure and soil erosion Table 5. The slope is the essential aspect of the surface as well as for internal soil water drainage as both characteristics play a significant role in the growth of the crop. The general slope suitability map is indicated in (S8 Fig) | | S8 Fig.tif, Crop slope suitability for South Africa, [Source, *South African Quaternary Catchments database,(* <https://doi.org/10.6084/m9.figshare.13179881>*), in ArcGIS 10.5* |
| Land Use Land cover-LULC of 2016 | | Knowledge of existing land use provides information about land availability. Land use data helps to identify the productivity of an area for a given cropping system. The land use and land cover (LULC) is a core information layer for a variety of scientific activities and administrative tasks in a given region. Understanding the proportion of land use is essential for the development of control measure, guide planners in making more informed decisions and achieving a balance between urban growth and preservation of the natural environment. The LULC map for cultivated areas in SA is shown in (S9 Fig) | | S9 Fig.tif, Crop production suitability map for land use land cover map for South Africa. [Source, *South African Quaternary Catchments database,(*[<https://doi.org/10.6084/m9.figshare.13179881>*g*](https://drive.google.com/drive/folders/1dwewc3rm_IPK9ylYOhkdpswG1dprMf-e?usp=sharing)*), in ArcGIS 10.5]* |
| Social and economic factors used to delineate land suitability maps for NUS. | | | | |
| Distance from road/accessibility | Land use is often also influenced by the ease of access to road networks for the transport of produce to the markets. Road networks play a vital role in remote areas, and the suitability analysis omits informal roads within farms though they play a crucial role in transportations of goods. S10 Fig, represent distance from road suitability map for SA. | | | S10 Fig.tif, Distance from road suitability map for South Africa, [Source, *South African Quaternary Catchments database,(*[[<https://doi.org/10.6084/m9.figshare.13179881>*g*](https://drive.google.com/drive/folders/1dwewc3rm_IPK9ylYOhkdpswG1dprMf-e?usp=sharing)*g*](https://drive.google.com/drive/folders/1dwewc3rm_IPK9ylYOhkdpswG1dprMf-e?usp=sharing)*), in ArcGIS 10.5]* |

References

1. Tibesigwa B, Visser M, Turpie J. Climate change and South Africa’s commercial farms: an assessment of impacts on specialised horticulture, crop, livestock and mixed farming systems. Environ Dev Sustain. 2017;

2. Aliber M, Cousins B. Livelihoods after Land Reform in South Africa. J Agrar Chang. 2013;

3. Smithers J, Schulze R. Long duration design rainfall estimates for South Africa. 2000;(811). Available from: http://www.wrc.org.za/Lists/Knowledge Hub Items/Attachments/7419/811-1-00_EXECUTIVE SUMMARY.pdf

4. Pereira LM. The future of the food system: Cases involving the private sector in South Africa. Sustain. 2013;

5. Pierrehumbert RT, Brogniez H, Roca R. On the relative humidity of the atmosphere. In: The Global Circulation of the Atmosphere. 2006.

6. Sage RF, Kubien DS. The temperature response of C3 and C4 photosynthesis. Plant, Cell and Environment. 2007.

7. Cannell MGR, Thornley JHM. Temperature and CO2 responses of leaf and canopy photosynthesis: A clarification using the non-rectangular hyperbola model of photosynthesis. Ann Bot. 1998;

8. Doorenbos J, Pruitt W. FAO Irrigation adn Drainage Paper 24: Crop Water Requirements. FAO Irrig Drain. 1977;

9. Raes D. AquaCrop training handbooks. Book I. Understanding AquaCrop. 2017;(July):1–59.

10. Raes D, Steduto P, Hsiao TC, Fereres E. Chapter 3: Calculation Procedures. Aquacrop Ref Man verion 40 [Internet]. 2012;(June):125. Available from: www.fao.org/publications

11. FAO. Land Degradation Assessment in Drylands. Mapping Land Use Systems at Global and Regional Scales for Land Degradation Assessment Analysis Version 1.1. FAO, GEF, Mec Glob la UNCCD, UNCCD, UNEP [Internet]. 2011;(February):84. Available from: http://120.52.51.13/www.fao.org/docrep/017/i3242e/i3242e.pdf

12. Schulze RE, Maharaj M. Moisture growing season. Production. 1978;1–3.

13. Consoli S, Vanella D. Mapping crop evapotranspiration by integrating vegetation indices into a soil water balance model. Agric Water Manag. 2014;

14. Heng LK, Hsiao T, Evett S, Howell T, Steduto P. Validating the FAO aquacrop model for irrigated and water defi cient field maize. Agron J. 2009;

15. Singh Rawat K, Kumar Singh S, Bala A, Szabó S. Estimation of crop evapotranspiration through spatial distributed crop coefficient in a semi-arid environment. Agric Water Manag. 2019;
