# Supplementary material for: Multi-criteria suitability analysis for neglected and underutilised crop species in South Africa
Source: PLoS One. 2021 Jan 19;16(1):e0244734. doi: 10.1371/journal.pone.0244734 (PMC7815157; doi:10.1371/journal.pone.0244734)
Supplement: Attachment — Submitted filename: Responses to reviewers_28_10_2020.docx [file pone.0244734.s013.docx]

**Responses to reviewer comments**

**Paper No. [PONE-D-20-15704] - [EMID:f47491bd0f719af3]**

**Title**: **Multi-criteria suitability analysis for neglected and underutilised crop species in South Africa**

Authors: H. Mugiyo^1*¶^, V.G.P. Chimonyo,^1¶^ M. Sibanda^1&^, R. Kunz^2&^, L. Nhamo^1 #a, 3#c^, C. Ramakgahlele Masemola^1^, C. Dalin^4&^, A.T. Modi^1^ and T. Mabhaudhi^1#a,2#b,¶^

^1^Centre for Transformative Agricultural and Food Systems, School of Agricultural, Earth & Environmental Sciences, University of KwaZulu-Natal, Pietermaritzburg, South Africa;

^2^Centre for Water Resources Research, School of Agricultural, Earth and Environmental Sciences, University of KwaZulu-Natal, Scottsville, South Africa;

^3^Water Research Commission of South Africa, 4 Daventry Street, Lynnwood Manor, Pretoria, South Africa;

^4^325 Central House Upper Woburn Place London

^#a^ Centre for Transformative Agricultural and Food Systems, School of Agricultural, Earth & Environmental Sciences, University of KwaZulu-Natal, Pietermaritzburg, South Africa;

^#b^Centre for Water Resources Research, School of Agricultural, Earth and Environmental Sciences, University of KwaZulu-Natal, Scottsville, South Africa;

^#c^Water Research Commission of South Africa, 4 Daventry Street, Lynnwood Manor, Pretoria, South Africa

*Correspondence: [mugiyoh@gmail.com](mailto:mugiyoh@gmail.com), (H.M)

**General response**: We want to take this opportunity to thank the Academic Editor for PLOS ONE, Dr Abel Chemura, as well as the two anonymous reviewers for their time spent in handling, reading and passing valuable comments that we used to improve our manuscript. We have reworked the manuscript according to each and every comment raised by reviewers, and we are confident that the changes made have improved the manuscript.

The data that support the findings of this study are publicly available. The paper contains all necessary instructions for independent researchers to obtain the data. Data is open access which means immediate, irrevocable and free online access to information products. All data is protected under Creative Commons (CC) license and Websites have been cited accordingly.

The data underlying the results presented in Fig 2, Fig 3, Fig 4, Fig 5, and Fig 6 in this study are available from the South African Quaternary Catchments database (https://doi.org/10.6084/m9.figshare.13179881), USGS FEWS NET (https://earlywarning.usgs.gov/fews), and from USGS Earth Explorer (<http://earthexplorer.usgs.gov>). In addition, the validation data gathered through field survey are available at (<https://doi.org/10.6084/m9.figshare.13179881>)*.* The shapefiles used in this study are available from the source of all spatial data in the National Geo-spatial Information (NGI) of South Africa, a component of Department of Rural Development and Land Reform (DRDLR).http://www.cdngiportal.co.za/cdngiportal/. The changes made in the manuscript are highlighted.

| **Editors comments** | **Author’s response and related revisions** |
| --- | --- |
| There is need for more broader context for the study beyond South Africa for an International Journal like PLoS which can be achieved by placing the importance of the study more globally. | We acknowledge the comment made by the editor and reviewers. To shift from a South African context and make it have a broader context, the following statements were added and/ or modified.  “The world is challenged by the need to feed a growing population with healthy food while minimising the negative impacts on the environment and adapting to changing climate (1). Despite the importance of smallholder agriculture to global food production and poverty reduction (2), in sub Saharan African (SSA) and Southeast Asia experience widespread food and nutrition insecurity (3)”.(See line 47-49)  The manuscript was contextualised in way to address the world challenges and relevant literature were cited for example  “Across the world, several research initiatives have looked into mechanisms that allow for stress adaptation within a range of NUS in SA (10), in Malaysia Peter et al. (11) research in the adoption of underutilised crops , Ebert from Taiwan (12) researched on potential of underutilized traditional vegetables and legume crops to contribute to food and nutritional security.” (See line 65– 69, 94-97, and 395-400)  In addition, the manuscript was contextualised in way to address the world challenges and relevant literature were cited for example  “Similar techniques have been used in agriculture by Musakwa identifying land suitable for agriculture (26), Kazemi et al. (27) for wheat , Zabihi et al. (28) for citrus, Kihoro et al. (29) for rice in Kenya, Benke and Pelizaro (30) for wheat and rye-grass, Malczewski et al. (31) for land suitability.”. (See line 94-97, and 395-400) |
| There is also need in the discussion for a more objective description of the limitations of the approach used in suitability modelling for readers to interpret the results more clearly. | We acknowledge the importance of discussing the limitations of the approach used in suitability modelling for readers to interpret the results more clearly. We have revised the discussion section include the caveats of the data as well as the approach used in this study. The following is an excerpt of the the changes we have made in that read.  “The consistency ratio was calculated as 0.05 (Table 3) and is considered as acceptable(24,79). To reduce the risk associated with over-fitting or noise modelling, nine thematic input layers were used by employing matrix pairwise comparison. The matrix pairwise comparison was obtained from different expects, and factor weights were calculated using a pairwise comparison matrix (Table 3).The accuracy of weights used is subjective as it depends on expert opinion; however, the results of the relative weights were used in land suitability evaluation because the Consistency Ratios were within the established acceptable limits (0.1)(79). The use of deterministic MCDA-AHP method is that assigning weights is subjective, and the setting of weights represent imprecise point estimates, and the process does not indicate error or confidence (30). Use of AHP methodology provides scope for combining expert opinion with measurements (38,80,81). Expert opinion weighted distance from the road with the lowest weight (Table 3), because the social-economic factor does not affect crop growth directly, but it influences the adoption of NUS by farmers. Accessibility to markets is highly influenced by road network because it affects markets. There other social-economic factors (availability to extension, markets, credits facilities) which can be included in MCDA to develop cropland suitability mapping which can try to model a farming system (82).Based on the analyses, there are potential environmental benefits to growing NUS in SA.  ” (See line 398 – 408) |
| The recommendations on the use of Lidar should also be put in the context of its cost-benefit ratio given the current levels of development of the technology, its cost and use for such analysis as proposed in this study. | We have added the following statement to contextualise the utility of LIDAR by future studies;  “One such sensor is LiDAR (Light Detection and Ranging) technology, which can provide 3D models of farmland (98). LiDAR technology can provide accurate maps of natural resources and farmlands for sustainable production of NUS in South Africa (99,100). High-resolution images in developing land suitability of NUS is of utmost importance in solving a land use land cover challenges, yet the process is often difficult, labour intensive and cost. The return on Investment (ROI) of using LiDAR in delineating areas suitable for NUS is low because several crops under class of NUS do not have well-structured value chain developing countries(102). The cost benefit of using LiDAR in smallholder farmers need to be evaluated to measure the efficiency of an investment (102).” (See line 576-587) |
| The recommendations are quite extensive and i suggest that you put these in prose paragraphs and not in bullets | We have rephrased and arranged the recommendations in prose paragraphs.  (See line 534-617) |
| Please ensure that your manuscript meets PLOS ONE's style requirements, including those for file naming. The PLOS ONE style templates can be found at | We have adopted the PLOS ONE's style requirements as well as the file naming system of the journal.  See the entire manuscript and the all the attached documents |
| We note that you have indicated that data from this study are available upon request. PLOS only allows data to be available upon request if there are legal or ethical restrictions on sharing data publicly. For more information on unacceptable data access restrictions, please see  [http://journals.plos.org/plosone/s/data-availability#loc-unacceptable-data-access-restrictions](https://protect-za.mimecast.com/s/aT0KCk5MXzfkL2Zrh87Qyg?domain=journals.plos.org).  a) If there are ethical or legal restrictions on sharing a de-identified data set, please explain them in detail (e.g., data contain potentially sensitive information, data are owned by a third-party organization, etc.) and who has imposed them (e.g., an ethics committee). Please also provide contact information for a data access committee, ethics committee, or other institutional body to which data requests may be sent.    b) If there are no restrictions, please upload the minimal anonymized data set necessary to replicate your study findings as either Supporting Information files or to a stable, public repository and provide us with the relevant URLs, DOIs, or accession numbers. For a list of acceptable repositories, please see [http://journals.plos.org/plosone/s/data-availability#loc-recommended-repositories](https://protect-za.mimecast.com/s/Smi_ClO6EAH19RDAcVBP64?domain=journals.plos.org). | We have acknowledged that the data underlying the results presented in this study are available from the South African Quaternary Catchments database (https://doi.org/10.6084/m9.figshare.13179881), USGS FEWS NET (https://earlywarning.usgs.gov/fews), and from USGS Earth Explorer (<http://earthexplorer.usgs.gov>)  and also will be availed upon request. |
| 3. PLOS requires an ORCID iD for the corresponding author in Editorial Manager on papers submitted after December 6th, 2016. Please ensure that you have an ORCID iD and that it is validated in Editorial Manager. To do this, go to ‘Update my Information’ (in the upper left-hand corner of the main menu), and click on the Fetch/Validate link next to the ORCID field. This will take you to the ORCID site and allow you to create a new iD or authenticate a pre-existing iD in Editorial Manager. Please see the following video for instructions on linking an ORCID iD to your Editorial Manager account: [https://www.youtube.com/watch?v=_xcclfuvtxQ](https://protect-za.mimecast.com/s/F_f8CmwXNBTp0yGAs33vPm?domain=youtube.com) | ORCID iD for the corresponding authors provided  The ORCID for the authors are as follows  H. Mugiyo: https://orcid.org/0000-0002-2121-357X  V.G.P. Chimonyo: https://orcid.org/0000-0001-9912-9848  M. Sibanda: <https://orcid.org/0000-0002-4589-7099>  R. Kunz  L. Nhamo: https://orcid.org/0000-0003-2944-1769  C. Ramakgahlele Masemola  C. Dalin  A.T. Modi1 and T. Mabhaudhi |
| 4. Please amend your list of authors on the manuscript to ensure that each author is linked to an affiliation. Authors’ affiliations should reflect the institution where the work was done (if authors moved subsequently, you can also list the new affiliation stating “current affiliation:….” as necessary). | We have amended the list of authors on the manuscript to ensure that each author is linked to curent institutions. |
| 5. We note you have included a table to which you do not refer in the text of your manuscript. Please ensure that you refer to Table 2 in your text; if accepted, production will need this reference to link the reader to the Table | Thank you, this is corrected, Table 2 was referred to text (See line 177) |
| 6.We note that [Supporting Information Figure 1, and Figure 2, 3, 4, 5 and 6 ] in your submission contain [map/satellite] images which may be copyrighted. All PLOS content is published under the Creative Commons Attribution License (CC BY 4.0), which means that the manuscript, images, and Supporting Information files will be freely available online, and any third party is permitted to access, download, copy, distribute, and use these materials in any way, even commercially, with proper attribution. For these reasons, we cannot publish previously copyrighted maps or satellite images created using proprietary data, such as Google software (Google Maps, Street View, and Earth). For more information, see our copyright guidelines: [http://journals.plos.org/plosone/s/licenses-and-copyright](https://protect-za.mimecast.com/s/wHdLCnZJ6DfXy9QKiv92Dx?domain=journals.plos.org). We require you to either (1) present written permission from the copyright holder to publish these figures specifically under the CC BY 4.0 license, or (2) remove the figures from your submission:  Please upload the completed Content Permission Form or other proof of granted permissions as an "Other" file with your submission. | We acknowledged. Data is open access which means immediate, irrevocable and free online access to information products. All data is protected under Creative Commons (CC) license and Websites have been cited accordingly  The shapefiles used in this study are available from the The source of all spatial data in SA is the National Geo-spatial Information (NGI), a component of Department of Rural Development and Land Reform (DRDLR).http://www.cdngiportal.co.za/cdngiportal/. |
| If you are unable to obtain permission from the original copyright holder to publish these figures under the CC BY 4.0 license or if the copyright holder’s requirements are incompatible with the CC BY 4.0 license, please either i) remove the figure or ii) supply a replacement figure that complies with the CC BY 4.0 license. Please check copyright information on all replacement figures and update the figure caption with source information. If applicable, please specify in the figure caption text when a figure is similar but not identical to the original image and is therefore for illustrative purposes only.  The following resources for replacing copyrighted map figures may be helpful:    USGS National Map Viewer (public domain): [http://viewer.nationalmap.gov/viewer/](https://protect-za.mimecast.com/s/vgELCqj8LJf14YGzcos7vS?domain=viewer.nationalmap.gov/)  The Gateway to Astronaut Photography of Earth (public domain): [http://eol.jsc.nasa.gov/sseop/clickmap/](https://protect-za.mimecast.com/s/BBXfCr07VKfrNQX1tmcenx?domain=eol.jsc.nasa.gov/)  Maps at the CIA (public domain): [https://www.cia.gov/library/publications/the-world-factbook/index.html](https://protect-za.mimecast.com/s/c0egCvgJLOSEBwo2FZIwXo?domain=cia.gov) and [https://www.cia.gov/library/publications/cia-maps-publications/index.html](https://protect-za.mimecast.com/s/-oDfCwjg7PfvOlXAFBoMnb?domain=cia.gov)  NASA Earth Observatory (public domain): [http://earthobservatory.nasa.gov/](https://protect-za.mimecast.com/s/npBXCxGjLQhLGEK9F3jvWU?domain=earthobservatory.nasa.gov/)  Landsat: [http://landsat.visibleearth.nasa.gov/](https://protect-za.mimecast.com/s/Qmk5Cy8k2Rt7jOE0FrWa9j?domain=landsat.visibleearth.nasa.gov/)  USGS EROS (Earth Resources Observatory and Science (EROS) Center) (public domain): [http://eros.usgs.gov/#](https://protect-za.mimecast.com/s/iPf_CzmlY0FGPWvxhpaIvx?domain=eros.usgs.gov)  Natural Earth (public domain): [http://www.naturalearthdata.com/](https://protect-za.mimecast.com/s/B-byCAnOKyfZmpMnfPY4kJ?domain=naturalearthdata.com/) | We acknowledged. Data is open access which means immediate, irrevocable and free online access to information products. All data is protected under Creative Commons (CC) license and Websites have been cited accordingly.  The data that support the findings of this study are publicly available. The paper contains all necessary instructions for independent researchers to obtain the data. The data underlying the results presented in this study are available from the South African Quaternary Catchments database (https://doi.org/10.6084/m9.figshare.13179881), USGS FEWS NET (https://earlywarning.usgs.gov/fews), and from USGS Earth Explorer (<http://earthexplorer.usgs.gov>). |
| Please include captions for your Supporting Information files at the end of your manuscript, , and update any in-text citations to match accordingly | We agree and thank you for observing this. All supporting Information files at the end of manuscript were aligned to PLOS ONE standards and reference were changed to Vancouver style |
| **Reviewer 1 comments** | **Author’s action** |
| 1. Is the manuscript technically sound, and do the data support the conclusions?  The manuscript must describe a technically sound piece of scientific research with data that supports the conclusions. Experiments must have been conducted rigorously, with appropriate controls, replication, and sample sizes. The conclusions must be drawn appropriately based on the data presented.  Reviewer #1: Partly  Reviewer #2: Yes  . | Thank you very much for the valuable comments that we used to improve the manuscript. We acknowledge the comment made by the reviewer. To shift from a South African context and make it have a broader context, the following statements were added and/ or modified.  The discussion was realigned to match objective description of the limitations of the approach used in suitability modelling for readers to interpret the results was explained more clearly. (See line 47-49, 65-71, 94-97 and 398-408) |
| 2. Has the statistical analysis been performed appropriately and rigorously?  Reviewer #1: Yes  Reviewer #2: Yes | Thank you for the encouraging comment |
| 3. Is the manuscript presented in an intelligible fashion and written in standard English?  PLOS ONE does not copyedit accepted manuscripts, so the language in submitted articles must be clear, correct, and unambiguous. Any typographical or grammatical errors should be corrected at revision, so please note any specific errors here.  Reviewer #1: No  Reviewer #2: No | We have addressed all grammatic errors and the responses are highlighted in the manuscript. The entire manuscript was edited and proof read by the 9 co-authors, and Grammarly premium was used to check errors |
| In abstract please mention the criteria with the highest impact on suitability  provide citation in SDGs in first paragraph | We have added a sentence in the abstract stating that:  “Rainfall was the most critical variable and criteria with the highest impact on land suitability of the NUS.”  See line 36-37 |
| More studies or literature on NUS beyond SA needed so that the study is grounded, because currently literature focuses on SA. Likewise a brief on MCDA techniques is needed and other studies on land suitability in SA for example in SA these two studies that use MCDA in SA are critical van Niekerk, A..;du Plessis, D..;Boonzaaier, I..;Spocter, M..;Ferreira, S..;Loots, L..;Donaldson, R., Development of a multi-criteria spatial planning support system for growth potential modelling in the Western Cape, South Africa. Land Use Policy, 2016. 50: p. 179-193. Musakwa, W., Identifying land suitable for agricultural land reform using GIS-MCDA in South Africa. Environment, Development and Sustainability, 2017: p. 1-19.  More relevant citations in Literature review such as is Jelokhani-Niaraki, M..;Malczewski, J., A group multicriteria spatial decision support system for parking site selection problem: A case study. Land Use Policy, 2015. 42: p. 492-508. 2. Jelokhani-Niaraki, M..;Malczewski, J., Decision complexity and consensus in Web-based spatial decision making: A case study of site selection problem using GIS and multicriteria analysis. Cities, 2015. 45(0): p. 60-  1. Malczewski, J., GIS and Multicriteria Decision Analysis. 1999, Toronto: John Wiley and Sons INC. 2. Malczewski, J., GIS Based multi criteria decision analysis: a survey of literature. International Journal of Geographical Information Science, 2006. 20(7): p. 703-726. 3. Malczewski, J., Ordered weighted averaging with fuzzy quantifiers: GIS-based multicriteria evaluation for land-use suitability analysis. International Journal of Applied Earth Observation and Geoinformation, 2006. 8(4): p. 270-277. 4. Malczewski, J., GIS-based multicriteria decision analysis: A survey of the literature. International Journal of Geographical Information Science, 2006. 20(7): p. 703-726. 5. Malczewski, J..;Rinner, C., Multicriteria decision analysis in geographic information science. 2015: Springer.  it’s crucial that these are cited | We have added more references including those suggested by the reviewer. |
| Line 91 more citations needed to justify statement | We added more citations needed to justify statement, the following statements were added and/ or modified.  “Similar techniques have been used in agriculture by Musakwa identifying land suitable for agriculture (26), Kazemi et al. for wheat (27), Zabihi et al. for citrus(28), Kamau et al. for rice in Kenya, , Benke and Pelizaro for wheat and rye-grass (29) (2010), Malczewski et al. for land suitability (30)”. (See line 94-97) |
| Line 97-100, its an assumption that NUS land suitability will induce production so rephrase statement | We agree and thank you for observing this. We have restructured the sentence as follows:  “Mapping NUS production potential zones in SA, will guide agriculturist why and where NUS can be promoted, assist decision-makers in formulating policies with a sustainable intensification concept and then the creation of markets for NUS, which will enhance food and nutrition security.” (See line 109-112) |
| Line 128 what is the date of the datasets and justify why these datasets. There are other better datasets from FAO, Copernucs that are far better than the current source Quaternary Catchments covering South Africa. A justification is needed | We agree and acknowledged the limitations of time frame. The time period is span from 1950-2000 covering 5 decades. In our discussion we discussed the limitations of datasets  “However, the interpretation of our results relative to climate change is limited by the fact that we used a historical data set (1950-2000). While this spans across half a decade, most of the extreme climate hazards have been observed in the last 30 years (1990 – present) (94). As such, future studies should focus on using data from global circulation models (GCMs) to inform climate change scenarios more specifically. However, the current maps remain useful for informing the areas that are currently suitable for NUS production, which is a first in South Africa.” (See line 496-502) |
| Line 163, remove error Line i65 what literature was used to weigh the criteria. can you provide more detail on how you did AHP.there is what is called the AHP calculator. | We agree and thank you for observing this. We have added source of AHP calculator used and provided in supplementary information . The sentence was restructured as follows:  “In this study, AHP calculator was used to calculate weights (50).The assignments of weights were based on information from literature, as well as the team’s local knowledge and expert consultation (soil scientist, GIS and remote sensing specialists from the University of KwaZulu Natal) (Table 3).” The calculator was derived from Kezima and the analysis shared in excel sheet. (See line 177-180) |
| line 185 remove error | Thank you for picking this. This has been corrected. |
| In the land suitability justify why certain areas are suitable or one.  What are some of the constraints to suitability, e.eg while cowpea is suitable in Gauteng, its highly urbanised province | We acknowledge the comment made by the reviewer. The constraints to suitability of cowpea in Gauteng highly urbanised province, the following statements were added and/ or modified.  “Cowpea sorghum and amaranths are highly suitable in areas which receives more than 500 mm person and most of these areas are highly urbanised especially in Gauteng province. Therefore, the opportunity cost of promoting NUS near urban areas might be affected by the land value near urban areas, then high valued crops like horticultural crops and dairy production with high market demand are more preferred by peri-urban farmers (9)”. (See line 428-432) |
| Maps showing the suitability per criteria is also needed | Maps showing the suitability per criteria has been given in the supplementary material. We thought maybe that it would not be necessary to add all 9 maps in the manuscript as some of this maps area already available in literature and also to show major results from MCDA-AHP. But the supplementary material has all single factor maps. |
| Change the collorsheme of the map so that they are visually please, i suggest red to green | Thank you for raising this issue of collorsheme on maps. All figures were formatted to match PLOS ONE standards |
| Remove the markers or dividers in the maps, remove title in map as this will be shown on caption, put legend on right hand corner see Musakwa 2017 Recommendations put them in one paragraph not in such detail in point form In concussion generalise what this means for Africa, and southern Africa | Thank you for raising this issue of c markers or dividers in the maps. All figures were formatted to match PLOS ONE standards |
| While revising your submission, please upload your figure files to the Preflight Analysis and Conversion Engine (PACE) digital diagnostic tool, [https://pacev2.apexcovantage.com/](https://protect-za.mimecast.com/s/NWYNCJZXWLfB49P1t2KxqS?domain=pacev2.apexcovantage.com/). PACE helps ensure that figures meet PLOS requirements. To use PACE, you must first register as a user. Registration is free. Then, login and navigate to the UPLOAD tab, where you will find detailed instructions on how to use the tool. If you encounter any issues or have any questions when using PACE, please email PLOS at [figures@plos.org](mailto:figures@plos.org). Please note that Supporting Information files do not need this step. | We acknowledge the importance of uploading figure files to the Pre-flight Analysis and Conversion Engine (PACE) to improve the quality of figures. All figures were formatted meet PLOS requirements |
| Please follow the below instructions to ensure that the figures & supporting information files in your submission adhere to PLOS ONE policy: a) Please upload each figure as a separate TIFF or EPS file. b) Name each of the figure files in numeric order with the format: Fig1.tif, Fig2.eps, and so on. c) Place figure captions in the manuscript text in read order, immediately following the paragraph where the figure is first cited. Do not include captions as part of the figure files or submit them in a separate document. | We acknowledge the importance of uploading figure files to the Pre-flight Analysis and Conversion Engine (PACE) to improve the quality of figures. All figures were formatted meet PLOS requirements.   - Figures were presented as separate file in .tif format, - Figure were numbered in numeric numbers and figure captions in the manuscript text were numbered correctly. |
| Please remove all Supporting Information and Supporting Information Figures from the end of your manuscript, as these should only be present in the separate Supporting Information file "Sup information for NUS WT.docx" | Thank for raising this issue, supporting information was presented separately . |
