# Supplementary material for: Multi-criteria suitability analysis for neglected and underutilised crop species in South Africa
Source: PLoS One. 2021 Jan 19;16(1):e0244734. doi: 10.1371/journal.pone.0244734 (PMC7815157; doi:10.1371/journal.pone.0244734)
Supplement: Peer review history

# Peer review: Decision Letter 0

**DOI:** 10.1371/journal.pone.0244734.r001

**Authors:** Abel Chemura

28 Jul 2020

PONE-D-20-15704

Multi-criteria suitability analysis for neglected and underutilised crop species in South Africa

PLOS ONE

Dear Dr. Mugiyo,

Thank you for submitting your manuscript to PLOS ONE. After careful consideration, we feel that it has merit but does not fully meet PLOS ONE’s publication criteria as it currently stands. Therefore, we invite you to submit a revised version of the manuscript that addresses the points raised during the review process.

==============================

There is need for more  broader context for the study beyond South Africa for an International Journal like PLoS which can be achieved by placing the importance of the study more globally. There is also need in the discussion for a more objective description of the limitations of the approach used in suitability modelling for readers to interpret the results more clearly. The recommendations on the use of Lidar should also be put in the context of its cost-benefit ratio given the current levels of development of the technology, its cost and use for such analysis as proposed in this study. The recommendations are quite extensive and i suggest that you put these in prose paragraphs and not in bullets.

==============================

Please submit your revised manuscript by Sep 11 2020 11:59PM. If you will need more time than this to complete your revisions, please reply to this message or contact the journal office at plosone@plos.org. When you're ready to submit your revision, log on to https://www.editorialmanager.com/pone/ and select the 'Submissions Needing Revision' folder to locate your manuscript file.

Please include the following items when submitting your revised manuscript:

- A rebuttal letter that responds to each point raised by the academic editor and reviewer(s). You should upload this letter as a separate file labeled 'Response to Reviewers'.
- A marked-up copy of your manuscript that highlights changes made to the original version. You should upload this as a separate file labeled 'Revised Manuscript with Track Changes'.
- An unmarked version of your revised paper without tracked changes. You should upload this as a separate file labeled 'Manuscript'.

If you would like to make changes to your financial disclosure, please include your updated statement in your cover letter. Guidelines for resubmitting your figure files are available below the reviewer comments at the end of this letter.

If applicable, we recommend that you deposit your laboratory protocols in protocols.io to enhance the reproducibility of your results. Protocols.io assigns your protocol its own identifier (DOI) so that it can be cited independently in the future. For instructions see: http://journals.plos.org/plosone/s/submission-guidelines#loc-laboratory-protocols

We look forward to receiving your revised manuscript.

Kind regards,

Abel Chemura

Academic Editor

PLOS ONE

Journal Requirements:

When submitting your revision, we need you to address these additional requirements.

1. Please ensure that your manuscript meets PLOS ONE's style requirements, including those for file naming. The PLOS ONE style templates can be found at

https://journals.plos.org/plosone/s/file?id=wjVg/PLOSOne_formatting_sample_main_body.pdf and

https://journals.plos.org/plosone/s/file?id=ba62/PLOSOne_formatting_sample_title_authors_affiliations.pdf

2.We note that you have indicated that data from this study are available upon request. PLOS only allows data to be available upon request if there are legal or ethical restrictions on sharing data publicly. For more information on unacceptable data access restrictions, please see http://journals.plos.org/plosone/s/data-availability#loc-unacceptable-data-access-restrictions.

In your revised cover letter, please address the following prompts:

a) If there are ethical or legal restrictions on sharing a de-identified data set, please explain them in detail (e.g., data contain potentially sensitive information, data are owned by a third-party organization, etc.) and who has imposed them (e.g., an ethics committee). Please also provide contact information for a data access committee, ethics committee, or other institutional body to which data requests may be sent.

b) If there are no restrictions, please upload the minimal anonymized data set necessary to replicate your study findings as either Supporting Information files or to a stable, public repository and provide us with the relevant URLs, DOIs, or accession numbers. For a list of acceptable repositories, please see http://journals.plos.org/plosone/s/data-availability#loc-recommended-repositories.

We will update your Data Availability statement on your behalf to reflect the information you provide.

3. PLOS requires an ORCID iD for the corresponding author in Editorial Manager on papers submitted after December 6th, 2016. Please ensure that you have an ORCID iD and that it is validated in Editorial Manager. To do this, go to ‘Update my Information’ (in the upper left-hand corner of the main menu), and click on the Fetch/Validate link next to the ORCID field. This will take you to the ORCID site and allow you to create a new iD or authenticate a pre-existing iD in Editorial Manager. Please see the following video for instructions on linking an ORCID iD to your Editorial Manager account: https://www.youtube.com/watch?v=_xcclfuvtxQ

4. Please amend your list of authors on the manuscript to ensure that each author is linked to an affiliation. Authors’ affiliations should reflect the institution where the work was done (if authors moved subsequently, you can also list the new affiliation stating “current affiliation:….” as necessary).

5. We note you have included a table to which you do not refer in the text of your manuscript. Please ensure that you refer to Table 2 in your text; if accepted, production will need this reference to link the reader to the Table.

6.We note that [Supporting Information Figure 1, and Figure 2, 3, 4, 5 and 6 ] in your submission contain [map/satellite] images which may be copyrighted. All PLOS content is published under the Creative Commons Attribution License (CC BY 4.0), which means that the manuscript, images, and Supporting Information files will be freely available online, and any third party is permitted to access, download, copy, distribute, and use these materials in any way, even commercially, with proper attribution. For these reasons, we cannot publish previously copyrighted maps or satellite images created using proprietary data, such as Google software (Google Maps, Street View, and Earth). For more information, see our copyright guidelines: http://journals.plos.org/plosone/s/licenses-and-copyright.

We require you to either (1) present written permission from the copyright holder to publish these figures specifically under the CC BY 4.0 license, or (2) remove the figures from your submission:

1.    You may seek permission from the original copyright holder of  [Supporting Information Figure 1, and Figure 2, 3, 4, 5 and 6 ] to publish the content specifically under the CC BY 4.0 license. 

We recommend that you contact the original copyright holder with the Content Permission Form (http://journals.plos.org/plosone/s/file?id=7c09/content-permission-form.pdf) and the following text:

“I request permission for the open-access journal PLOS ONE to publish XXX under the Creative Commons Attribution License (CCAL) CC BY 4.0 (http://creativecommons.org/licenses/by/4.0/). Please be aware that this license allows unrestricted use and distribution, even commercially, by third parties. Please reply and provide explicit written permission to publish XXX under a CC BY license and complete the attached form.”

Please upload the completed Content Permission Form or other proof of granted permissions as an "Other" file with your submission.

In the figure caption of the copyrighted figure, please include the following text: “Reprinted from [ref] under a CC BY license, with permission from [name of publisher], original copyright [original copyright year].”

2.    If you are unable to obtain permission from the original copyright holder to publish these figures under the CC BY 4.0 license or if the copyright holder’s requirements are incompatible with the CC BY 4.0 license, please either i) remove the figure or ii) supply a replacement figure that complies with the CC BY 4.0 license. Please check copyright information on all replacement figures and update the figure caption with source information. If applicable, please specify in the figure caption text when a figure is similar but not identical to the original image and is therefore for illustrative purposes only.

The following resources for replacing copyrighted map figures may be helpful:

USGS National Map Viewer (public domain): http://viewer.nationalmap.gov/viewer/

The Gateway to Astronaut Photography of Earth (public domain): http://eol.jsc.nasa.gov/sseop/clickmap/

Maps at the CIA (public domain): https://www.cia.gov/library/publications/the-world-factbook/index.html and https://www.cia.gov/library/publications/cia-maps-publications/index.html

NASA Earth Observatory (public domain): http://earthobservatory.nasa.gov/

Landsat: http://landsat.visibleearth.nasa.gov/

USGS EROS (Earth Resources Observatory and Science (EROS) Center) (public domain): http://eros.usgs.gov/#

Natural Earth (public domain): http://www.naturalearthdata.com/

7. Please include captions for your Supporting Information files at the end of your manuscript, and update any in-text citations to match accordingly. Please see our Supporting Information guidelines for more information: http://journals.plos.org/plosone/s/supporting-information.

[Note: HTML markup is below. Please do not edit.]

Reviewers' comments:

Reviewer's Responses to Questions

**Comments to the Author**

1. Is the manuscript technically sound, and do the data support the conclusions?

The manuscript must describe a technically sound piece of scientific research with data that supports the conclusions. Experiments must have been conducted rigorously, with appropriate controls, replication, and sample sizes. The conclusions must be drawn appropriately based on the data presented. 

Reviewer #1: Partly

Reviewer #2: Yes

**********

2. Has the statistical analysis been performed appropriately and rigorously? 

Reviewer #1: Yes

Reviewer #2: Yes

**********

3. Have the authors made all data underlying the findings in their manuscript fully available?

The PLOS Data policy requires authors to make all data underlying the findings described in their manuscript fully available without restriction, with rare exception (please refer to the Data Availability Statement in the manuscript PDF file). The data should be provided as part of the manuscript or its supporting information, or deposited to a public repository. For example, in addition to summary statistics, the data points behind means, medians and variance measures should be available. If there are restrictions on publicly sharing data—e.g. participant privacy or use of data from a third party—those must be specified.

Reviewer #1: Yes

Reviewer #2: Yes

**********

4. Is the manuscript presented in an intelligible fashion and written in standard English?

PLOS ONE does not copyedit accepted manuscripts, so the language in submitted articles must be clear, correct, and unambiguous. Any typographical or grammatical errors should be corrected at revision, so please note any specific errors here.

Reviewer #1: No

Reviewer #2: No

**********

5. Review Comments to the Author

Please use the space provided to explain your answers to the questions above. You may also include additional comments for the author, including concerns about dual publication, research ethics, or publication ethics. (Please upload your review as an attachment if it exceeds 20,000 characters)

Reviewer #1: I commend the authors on this study, it is crucial in these times of climate change and the need for food security. I enjoyed reading the paper and i believe it ads on to knowledge. However for the paper to be published that need to adress the issues below

In abstract please mention the criteria with the highest impact on suitability

provide citation in SDGs in first paragraph

More studies or literature on NUS beyond SA needed so that the study is grounded, because currently literature focuses on SA. Likewise a brief on MCDA techniseqs is needed and other studies on land suitability in SA for example in SA these two studies that use MCDA in SA are critical van Niekerk, A..;du Plessis, D..;Boonzaaier, I..;Spocter, M..;Ferreira, S..;Loots, L..;Donaldson, R., Development of a multi-criteria spatial planning support system for growth potential modelling in the Western Cape, South Africa. Land Use Policy, 2016. 50: p. 179-193.

Musakwa, W., Identifying land suitable for agricultural land reform using GIS-MCDA in South Africa. Environment, Development and Sustainability, 2017: p. 1-19.

More relavant citaiaons in Literaure review such as is Jelokhani-Niaraki, M..;Malczewski, J., A group multicriteria spatial decision support system for parking site selection problem: A case study. Land Use Policy, 2015. 42: p. 492-508.

2. Jelokhani-Niaraki, M..;Malczewski, J., Decision complexity and consensus in Web-based spatial decision making: A case study of site selection problem using GIS and multicriteria analysis. Cities, 2015. 45(0): p. 60-

1. Malczewski, J., GIS and Multicriteria Decision Analysis. 1999, Toronto: John Wiley and Sons INC.

2. Malczewski, J., GIS Based multi criteria decision analysis:a survey of literature. International Journal of Geographical Information Science, 2006. 20(7): p. 703-726.

3. Malczewski, J., Ordered weighted averaging with fuzzy quantifiers: GIS-based multicriteria evaluation for land-use suitability analysis. International Journal of Applied Earth Observation and Geoinformation, 2006. 8(4): p. 270-277.

4. Malczewski, J., GIS-based multicriteria decision analysis: A survey of the literature. International Journal of Geographical Information Science, 2006. 20(7): p. 703-726.

5. Malczewski, J..;Rinner, C., Multicriteria decision analysis in geographic information science. 2015: Springer.

its crucial that these are cited

Line 91 more citations needed to justify statement

Line 97-100, its an assumption that NUS land suitability will induce production so rephrase statement

Line 128 what is the date of the datasets and justify why these datasets.There are other better datasets from FAO, Copernucs that are far better than the current source Quaternary Catchments covering South Africa.A justification is needed

Line 163, remove error

Line i65 what literature was used to weigh the criteria. can you provide more detail on how you did AHP.there is what is called the AHP calculator.

line 185 remove error

Line 215 FAO citation needed

Remove Error! Reference source not in whole document

Maps showing the suitability per criteria is also needed

In the land suitability justify why certain areas are suitable or one

Change the collorsheme of the map so that they are visually please, i suggest red to green

What are some of the constraints to suitability, e.eg while cowpea is suitable in gauteng, its highly urbanised province

Remove the markers or dividers in the maps, remove title in map as this will be shown on caption, put legend on right hand corner see Musakwa 2017

Recommendations put them in one paragraph not in such detail in point form

In concussion generalise what this means for Africa, and southern Africa

Reviewer #2: The research is quite interesting. The research gap has been highlighted. Method is clear. However a number of editorial issues should be addressed.

EDITORIAL ISSUES

• REFERENCE STYLES MIXED

• A NUMBER OF REFERENCES NOT FOUND

• Table 1 repeated

**********

6. PLOS authors have the option to publish the peer review history of their article (what does this mean?). If published, this will include your full peer review and any attached files.

If you choose “no”, your identity will remain anonymous but your review may still be made public.

**Do you want your identity to be public for this peer review?** For information about this choice, including consent withdrawal, please see our Privacy Policy.

Reviewer #1: No

Reviewer #2: No

[NOTE: If reviewer comments were submitted as an attachment file, they will be attached to this email and accessible via the submission site. Please log into your account, locate the manuscript record, and check for the action link "View Attachments". If this link does not appear, there are no attachment files.]

While revising your submission, please upload your figure files to the Preflight Analysis and Conversion Engine (PACE) digital diagnostic tool, https://pacev2.apexcovantage.com/. PACE helps ensure that figures meet PLOS requirements. To use PACE, you must first register as a user. Registration is free. Then, login and navigate to the UPLOAD tab, where you will find detailed instructions on how to use the tool. If you encounter any issues or have any questions when using PACE, please email PLOS at figures@plos.org. Please note that Supporting Information files do not need this step.

# Author comment: Author response to Decision Letter 0

**DOI:** 10.1371/journal.pone.0244734.r002

23 Nov 2020

Responses to reviewer comments

Paper No. [PONE-D-20-15704] - [EMID:f47491bd0f719af3]

Title: Multi-criteria suitability analysis for neglected and underutilised crop species in South Africa

Authors: H. Mugiyo1*¶, V.G.P. Chimonyo,1¶ M. Sibanda1&, R. Kunz2&, L. Nhamo1 #a, 3#b, C. Ramakgahlele Masemola1, C. Dalin4&, A.T. Modi1 and T. Mabhaudhi1#a,2#b,¶

General response: We want to take this opportunity to thank the Academic Editor for PLOS ONE, Dr Abel Chemura, as well as the two anonymous reviewers for their time spent in handling, reading and passing valuable comments that we used to improve our manuscript. We have reworked the manuscript according to each and every comment raised by reviewers, and we are confident that the changes made have improved the manuscript. 

The data that support the findings of this study are publicly available. The paper contains all necessary instructions for independent researchers to obtain the data. Data is open access which means immediate, irrevocable and free online access to information products. All data is protected under Creative Commons (CC) license and Websites have been cited accordingly.

The changes made in the manuscript are highlighted.

Editors comments Author’s response and related revisions

There is need for more broader context for the study beyond South Africa for an International Journal like PLoS which can be achieved by placing the importance of the study more globally. We acknowledge the comment made by the editor and reviewers. To shift from a South African context and make it have a broader context, the following statements were added and/ or modified.

“The world is challenged by the need to feed a growing population with healthy food while minimising the negative impacts on the environment and adapting to changing climate (1). Despite the importance of smallholder agriculture to global food production and poverty reduction (2), in sub Saharan African (SSA) and Southeast Asia experience widespread food and nutrition insecurity (3)”.(See line 38 – 42)

The manuscript was contextualised in way to address the world challenges and relevant literature were cited for example

“Across the world, several research initiatives have looked into mechanisms that allow for stress adaptation within a range of NUS in SA (10), in Malaysia Peter et al. (11) research in the adoption of underutilised crops , Ebert from Taiwan (12) researched on potential of underutilized traditional vegetables and legume crops to contribute to food and nutritional security.” (See line 57 – 60)

In addition, the manuscript was contextualised in way to address the world challenges and relevant literature were cited for example

“Similar techniques have been used in agriculture by Musakwa identifying land suitable for agriculture (26), Kazemi et al. (27) for wheat , Zabihi et al. (28) for citrus, Kihoro et al. (29) for rice in Kenya, Benke and Pelizaro (30) for wheat and rye-grass, Malczewski et al. (31) for land suitability.”. (See line 83-86, and 327-329)

There is also need in the discussion for a more objective description of the limitations of the approach used in suitability modelling for readers to interpret the results more clearly We acknowledge the importance of discussing the limitations of the approach used in suitability modelling for readers to interpret the results more clearly, the following statements were added and/ or modified.

“The consistency ratio was calculated as 0.05 (Table 3) and is considered as acceptable(24,79). To reduce the risk associated with over-fitting or noise modelling, nine thematic input layers were used by employing matrix pairwise comparison. The matrix pairwise comparison was obtained from different expects, and factor weights were calculated using a pairwise comparison matrix (Table 3).The accuracy of weights used is subjective as it depends on expert opinion; however, the results of the relative weights were used in land suitability evaluation because the Consistency Ratios were within the established acceptable limits (0.1)(79). The use of deterministic MCDA-AHP method is that assigning weights is subjective, and the setting of weights represent imprecise point estimates, and the process does not indicate error or confidence (30). Use of AHP methodology provides scope for combining expert opinion with measurements (38,80,81). Expert opinion weighted distance from the road with the lowest weight (Table 3), because the social-economic factor does not affect crop growth directly, but it influences the adoption of NUS by farmers. Accessibility to markets is highly influenced by road network because it affects markets. There other social-economic factors (availability to extension, markets, credits facilities) which can be included in MCDA to develop cropland suitability mapping which can try to model a farming system (82).Based on the analyses, there are potential environmental benefits to growing NUS in SA.

” (See line 330 – 344)

The recommendations on the use of Lidar should also be put in the context of its cost-benefit ratio given the current levels of development of the technology, its cost and use for such analysis as proposed in this study. We acknowledged the importance of cost-benefit ratio of LiDAR to NUS. The following statements were added 

“One such sensor is LiDAR (Light Detection and Ranging) technology, which can provide 3D models of farmland (98). LiDAR technology can provide accurate maps of natural resources and farmlands for sustainable production of NUS in South Africa (99,100). High-resolution images in developing land suitability of NUS is of utmost importance in solving a land use land cover challenges, yet the process is often difficult, labour intensive and cost. The return on Investment (ROI) of using LiDAR in delineating areas suitable for NUS is low because several crops under class of NUS do not have well-structured value chain developing countries(102). The cost benefit of using LiDAR in smallholder farmers need to be evaluated to measure the efficiency of an investment (102).” (See line 478-482)

The recommendations are quite extensive and i suggest that you put these in prose paragraphs and not in bullets Thank you for bringing this. The recommendations were rephrased and put these in prose paragraphs. (See line 448-500)

Please ensure that your manuscript meets PLOS ONE's style requirements, including those for file naming. The PLOS ONE style templates can be found at

 Thank you very much for the valuable comments that we used to improve the manuscript. We addressed all the comments, and the responses are highlighted in the manuscript to meet PLOS ONE's style requirements.

We note that you have indicated that data from this study are available upon request. PLOS only allows data to be available upon request if there are legal or ethical restrictions on sharing data publicly. For more information on unacceptable data access restrictions, please see

http://journals.plos.org/plosone/s/data-availability#loc-unacceptable-data-access-restrictions.

a) If there are ethical or legal restrictions on sharing a de-identified data set, please explain them in detail (e.g., data contain potentially sensitive information, data are owned by a third-party organization, etc.) and who has imposed them (e.g., an ethics committee). Please also provide contact information for a data access committee, ethics committee, or other institutional body to which data requests may be sent.

b) If there are no restrictions, please upload the minimal anonymized data set necessary to replicate your study findings as either Supporting Information files or to a stable, public repository and provide us with the relevant URLs, DOIs, or accession numbers. For a list of acceptable repositories, please see http://journals.plos.org/plosone/s/data-availability#loc-recommended-repositories.

 We acknowledged. Data is open access which means immediate, irrevocable and free online access to information products. All data is protected under Creative Commons (CC) license and Websites have been cited accordingly

3. PLOS requires an ORCID iD for the corresponding author in Editorial Manager on papers submitted after December 6th, 2016. Please ensure that you have an ORCID iD and that it is validated in Editorial Manager. To do this, go to ‘Update my Information’ (in the upper left-hand corner of the main menu), and click on the Fetch/Validate link next to the ORCID field. This will take you to the ORCID site and allow you to create a new iD or authenticate a pre-existing iD in Editorial Manager. Please see the following video for instructions on linking an ORCID iD to your Editorial Manager account: https://www.youtube.com/watch?v=_xcclfuvtxQ

 ORCID iD for the corresponding authors provided

4. Please amend your list of authors on the manuscript to ensure that each author is linked to an affiliation. Authors’ affiliations should reflect the institution where the work was done (if authors moved subsequently, you can also list the new affiliation stating “current affiliation:….” as necessary).

 Authors’ affiliations reflect the institutions

5. We note you have included a table to which you do not refer in the text of your manuscript. Please ensure that you refer to Table 2 in your text; if accepted, production will need this reference to link the reader to the Table Thank you, this is corrected, table 2 was referred to text (See line 160)

6.We note that [Supporting Information Figure 1, and Figure 2, 3, 4, 5 and 6 ] in your submission contain [map/satellite] images which may be copyrighted. All PLOS content is published under the Creative Commons Attribution License (CC BY 4.0), which means that the manuscript, images, and Supporting Information files will be freely available online, and any third party is permitted to access, download, copy, distribute, and use these materials in any way, even commercially, with proper attribution. For these reasons, we cannot publish previously copyrighted maps or satellite images created using proprietary data, such as Google software (Google Maps, Street View, and Earth). For more information, see our copyright guidelines: http://journals.plos.org/plosone/s/licenses-and-copyright. We require you to either (1) present written permission from the copyright holder to publish these figures specifically under the CC BY 4.0 license, or (2) remove the figures from your submission:

Please upload the completed Content Permission Form or other proof of granted permissions as an "Other" file with your submission.

 We acknowledged. Data is open access which means immediate, irrevocable and free online access to information products. All data is protected under Creative Commons (CC) license and Websites have been cited accordingly

If you are unable to obtain permission from the original copyright holder to publish these figures under the CC BY 4.0 license or if the copyright holder’s requirements are incompatible with the CC BY 4.0 license, please either i) remove the figure or ii) supply a replacement figure that complies with the CC BY 4.0 license. Please check copyright information on all replacement figures and update the figure caption with source information. If applicable, please specify in the figure caption text when a figure is similar but not identical to the original image and is therefore for illustrative purposes only.

The following resources for replacing copyrighted map figures may be helpful:

USGS National Map Viewer (public domain): http://viewer.nationalmap.gov/viewer/

The Gateway to Astronaut Photography of Earth (public domain): http://eol.jsc.nasa.gov/sseop/clickmap/

Maps at the CIA (public domain): https://www.cia.gov/library/publications/the-world-factbook/index.html and https://www.cia.gov/library/publications/cia-maps-publications/index.html

NASA Earth Observatory (public domain): http://earthobservatory.nasa.gov/

Landsat: http://landsat.visibleearth.nasa.gov/

USGS EROS (Earth Resources Observatory and Science (EROS) Center) (public domain): http://eros.usgs.gov/#

Natural Earth (public domain): http://www.naturalearthdata.com/

 We acknowledged. Data is open access which means immediate, irrevocable and free online access to information products. All data is protected under Creative Commons (CC) license and Websites have been cited accordingly.

The data that support the findings of this study are publicly available. The paper contains all necessary instructions for independent researchers to obtain the data.

Please include captions for your Supporting Information files at the end of your manuscript, , and update any in-text citations to match accordingly We agree and thank you for observing this. All supporting Information files at the end of manuscript were aligned to PLOS ONE standards and reference were changed to Vancouver style

Reviewer 1 comments Author’s action

1. Is the manuscript technically sound, and do the data support the conclusions?

The manuscript must describe a technically sound piece of scientific research with data that supports the conclusions. Experiments must have been conducted rigorously, with appropriate controls, replication, and sample sizes. The conclusions must be drawn appropriately based on the data presented. 

Reviewer #1: Partly

Reviewer #2: Yes

. Thank you very much for the valuable comments that we used to improve the manuscript. We acknowledge the comment made by the reviewer. To shift from a South African context and make it have a broader context, the following statements were added and/ or modified.

The discussion was realigned to match objective description of the limitations of the approach used in suitability modelling for readers to interpret the results was explained more clearly. (See line 38-42, 57-60, 83-86 and 330-344)

2. Has the statistical analysis been performed appropriately and rigorously? 

Reviewer #1: Yes

Reviewer #2: Yes

 Thank you for the encouraging comment

3. Is the manuscript presented in an intelligible fashion and written in standard English?

PLOS ONE does not copyedit accepted manuscripts, so the language in submitted articles must be clear, correct, and unambiguous. Any typographical or grammatical errors should be corrected at revision, so please note any specific errors here.

Reviewer #1: No

Reviewer #2: No

 Thank you very much for the valuable comments that we used to improve the manuscript. We addressed all grammatic errors and the responses are highlighted in the manuscript. The entire manuscript was edited and proof read by 9 co-authors, and Grammarly premium was used to check errors 

In abstract please mention the criteria with the highest impact on suitability

provide citation in SDGs in first paragraph Several neglected and underutilised species (NUS) can provide solutions for climate change adaptation and creating a Zero Hunger world, the Sustainable Development Goal 2. These NUS are drought- and heat stress-tolerant, making them ideal for improving marginalised cropping systems in drought-prone areas for South Africa (SA). In rainfed NUS production, rainfall is the major limiting factor. (See line 16-17)

More studies or literature on NUS beyond SA needed so that the study is grounded, because currently literature focuses on SA. Likewise a brief on MCDA techniques is needed and other studies on land suitability in SA for example in SA these two studies that use MCDA in SA are critical van Niekerk, A..;du Plessis, D..;Boonzaaier, I..;Spocter, M..;Ferreira, S..;Loots, L..;Donaldson, R., Development of a multi-criteria spatial planning support system for growth potential modelling in the Western Cape, South Africa. Land Use Policy, 2016. 50: p. 179-193.

Musakwa, W., Identifying land suitable for agricultural land reform using GIS-MCDA in South Africa. Environment, Development and Sustainability, 2017: p. 1-19.

More relevant citations in Literature review such as is Jelokhani-Niaraki, M..;Malczewski, J., A group multicriteria spatial decision support system for parking site selection problem: A case study. Land Use Policy, 2015. 42: p. 492-508.

2. Jelokhani-Niaraki, M..;Malczewski, J., Decision complexity and consensus in Web-based spatial decision making: A case study of site selection problem using GIS and multicriteria analysis. Cities, 2015. 45(0): p. 60-

1. Malczewski, J., GIS and Multicriteria Decision Analysis. 1999, Toronto: John Wiley and Sons INC.

2. Malczewski, J., GIS Based multi criteria decision analysis: a survey of literature. International Journal of Geographical Information Science, 2006. 20(7): p. 703-726.

3. Malczewski, J., Ordered weighted averaging with fuzzy quantifiers: GIS-based multicriteria evaluation for land-use suitability analysis. International Journal of Applied Earth Observation and Geoinformation, 2006. 8(4): p. 270-277.

4. Malczewski, J., GIS-based multicriteria decision analysis: A survey of the literature. International Journal of Geographical Information Science, 2006. 20(7): p. 703-726.

5. Malczewski, J..;Rinner, C., Multicriteria decision analysis in geographic information science. 2015: Springer.

it’s crucial that these are cited Thank you, the references have been added 

Line 91 more citations needed to justify statement We added more citations needed to justify statement, the following statements were added and/ or modified.

“Similar techniques have been used in agriculture by Musakwa identifying land suitable for agriculture (26), Kazemi et al. for wheat (27), Zabihi et al. for citrus(28), Kamau et al. for rice in Kenya, , Benke and Pelizaro for wheat and rye-grass (29) (2010), Malczewski et al. for land suitability (30)”. (See line 83-86)

Line 97-100, its an assumption that NUS land suitability will induce production so rephrase statement

 We agree and thank you for observing this. We have restructured the sentence as follows:

“Mapping NUS production potential zones in SA, will guide agriculturist why and where NUS can be promoted, assist decision-makers in formulating policies with a sustainable intensification concept and then the creation of markets for NUS, which will enhance food and nutrition security.” (See line 97-100)

Line 128 what is the date of the datasets and justify why these datasets. There are other better datasets from FAO, Copernucs that are far better than the current source Quaternary Catchments covering South Africa. A justification is needed

 We agree and acknowledged the limitations of time frame. The time period is span from 1950-2000 covering 5 decades. In our discussion we discussed the limitations of datasets

“However, the interpretation of our results relative to climate change is limited by the fact that we used a historical data set (1950-2000). While this spans across half a decade, most of the extreme climate hazards have been observed in the last 30 years (1990 – present) (94). As such, future studies should focus on using data from global circulation models (GCMs) to inform climate change scenarios more specifically. However, the current maps remain useful for informing the areas that are currently suitable for NUS production, which is a first in South Africa.” (See line 409-415)

Line 163, remove error

Line i65 what literature was used to weigh the criteria. can you provide more detail on how you did AHP.there is what is called the AHP calculator. We agree and thank you for observing this. We have added source of AHP calculator used and provided in supplementary information . The sentence was restructured as follows:

“In this study, AHP calculator was used to calculate weights (50).The assignments of weights were based on information from literature, as well as the team’s local knowledge and expert consultation (soil scientist, GIS and remote sensing specialists from the University of KwaZulu Natal) (Table 3).” The calculator was derived from Kezima and the analysis shared in excel sheet. (See line 160-163)

line 185 remove error Thank you for picking this. This has been corrected. 

In the land suitability justify why certain areas are suitable or one.

What are some of the constraints to suitability, e.eg while cowpea is suitable in Gauteng, its highly urbanised province We acknowledge the comment made by the reviewer. The constraints to suitability of cowpea in Gauteng highly urbanised province, the following statements were added and/ or modified.

“Cowpea sorghum and amaranths are highly suitable in areas which receives more than 500 mm person and most of these areas are highly urbanised especially in Gauteng province. Therefore, the opportunity cost of promoting NUS near urban areas might be affected by the land value near urban areas, then high valued crops like horticultural crops and dairy production with high market demand are more preferred by peri-urban farmers (9)”. (See line 385-387)

Maps showing the suitability per criteria is also needed Maps showing the suitability per criteria has been given in the supplementary material. We thought maybe that it would not be necessary to add all 9 maps in the manuscript as some of this maps area already available in literature and also to show major results from MCDA-AHP. But the supplementary material has all single factor maps.

Change the collorsheme of the map so that they are visually please, i suggest red to green

 Thank you for raising this issue of collorsheme on maps. All figures were formatted to match PLOS ONE standards

Remove the markers or dividers in the maps, remove title in map as this will be shown on caption, put legend on right hand corner see Musakwa 2017

Recommendations put them in one paragraph not in such detail in point form

In concussion generalise what this means for Africa, and southern Africa

 Thank you for raising this issue of c markers or dividers in the maps. All figures were formatted to match PLOS ONE standards

While revising your submission, please upload your figure files to the Preflight Analysis and Conversion Engine (PACE) digital diagnostic tool, https://pacev2.apexcovantage.com/. PACE helps ensure that figures meet PLOS requirements. To use PACE, you must first register as a user. Registration is free. Then, login and navigate to the UPLOAD tab, where you will find detailed instructions on how to use the tool. If you encounter any issues or have any questions when using PACE, please email PLOS at figures@plos.org. Please note that Supporting Information files do not need this step. We acknowledge the importance of uploading figure files to the Pre-flight Analysis and Conversion Engine (PACE) to improve the quality of figures. All figures were formatted meet PLOS requirements

Attachment Submitted filename: Responses to reviewers_28_10_2020.docx Click here for additional data file.

# Peer review: Decision Letter 1

**DOI:** 10.1371/journal.pone.0244734.r003

**Authors:** Abel Chemura

16 Dec 2020

Multi-criteria suitability analysis for neglected and underutilised crop species in South Africa

PONE-D-20-15704R1

Dear Dr. Mugiyo,

We’re pleased to inform you that your manuscript has been judged scientifically suitable for publication and will be formally accepted for publication once it meets all outstanding technical requirements.

Within one week, you’ll receive an e-mail detailing the required amendments. When these have been addressed, you’ll receive a formal acceptance letter and your manuscript will be scheduled for publication.

An invoice for payment will follow shortly after the formal acceptance. To ensure an efficient process, please log into Editorial Manager at http://www.editorialmanager.com/pone/, click the 'Update My Information' link at the top of the page, and double check that your user information is up-to-date. If you have any billing related questions, please contact our Author Billing department directly at authorbilling@plos.org.

If your institution or institutions have a press office, please notify them about your upcoming paper to help maximize its impact. If they’ll be preparing press materials, please inform our press team as soon as possible -- no later than 48 hours after receiving the formal acceptance. Your manuscript will remain under strict press embargo until 2 pm Eastern Time on the date of publication. For more information, please contact onepress@plos.org.

Kind regards,

Abel Chemura

Academic Editor

PLOS ONE

Additional Editor Comments (optional):

Reviewers' comments:

Reviewer's Responses to Questions

**Comments to the Author**

1. If the authors have adequately addressed your comments raised in a previous round of review and you feel that this manuscript is now acceptable for publication, you may indicate that here to bypass the “Comments to the Author” section, enter your conflict of interest statement in the “Confidential to Editor” section, and submit your "Accept" recommendation.

Reviewer #1: All comments have been addressed

Reviewer #2: All comments have been addressed

**********

2. Is the manuscript technically sound, and do the data support the conclusions?

The manuscript must describe a technically sound piece of scientific research with data that supports the conclusions. Experiments must have been conducted rigorously, with appropriate controls, replication, and sample sizes. The conclusions must be drawn appropriately based on the data presented. 

Reviewer #1: Yes

Reviewer #2: Yes

**********

3. Has the statistical analysis been performed appropriately and rigorously? 

Reviewer #1: Yes

Reviewer #2: (No Response)

**********

4. Have the authors made all data underlying the findings in their manuscript fully available?

The PLOS Data policy requires authors to make all data underlying the findings described in their manuscript fully available without restriction, with rare exception (please refer to the Data Availability Statement in the manuscript PDF file). The data should be provided as part of the manuscript or its supporting information, or deposited to a public repository. For example, in addition to summary statistics, the data points behind means, medians and variance measures should be available. If there are restrictions on publicly sharing data—e.g. participant privacy or use of data from a third party—those must be specified.

Reviewer #1: Yes

Reviewer #2: Yes

**********

5. Is the manuscript presented in an intelligible fashion and written in standard English?

PLOS ONE does not copyedit accepted manuscripts, so the language in submitted articles must be clear, correct, and unambiguous. Any typographical or grammatical errors should be corrected at revision, so please note any specific errors here.

Reviewer #1: Yes

Reviewer #2: Yes

**********

6. Review Comments to the Author

Please use the space provided to explain your answers to the questions above. You may also include additional comments for the author, including concerns about dual publication, research ethics, or publication ethics. (Please upload your review as an attachment if it exceeds 20,000 characters)

Reviewer #1: N/A

comments have been addressed to my satisfaction, hence in my opinion no need for further corrections

Reviewer #2: (No Response)

**********

7. PLOS authors have the option to publish the peer review history of their article (what does this mean?). If published, this will include your full peer review and any attached files.

If you choose “no”, your identity will remain anonymous but your review may still be made public.

**Do you want your identity to be public for this peer review?** For information about this choice, including consent withdrawal, please see our Privacy Policy.

Reviewer #1: No

Reviewer #2: No

# Editor report: Acceptance letter

**DOI:** 10.1371/journal.pone.0244734.r004

**Authors:** Abel Chemura

29 Dec 2020

PONE-D-20-15704R1 

Multi-criteria suitability analysis for neglected and underutilised crop species in South Africa 

Dear Dr. Mugiyo:

I'm pleased to inform you that your manuscript has been deemed suitable for publication in PLOS ONE. Congratulations! Your manuscript is now with our production department. 

If your institution or institutions have a press office, please let them know about your upcoming paper now to help maximize its impact. If they'll be preparing press materials, please inform our press team within the next 48 hours. Your manuscript will remain under strict press embargo until 2 pm Eastern Time on the date of publication. For more information please contact onepress@plos.org.

If we can help with anything else, please email us at plosone@plos.org. 

Thank you for submitting your work to PLOS ONE and supporting open access. 

Kind regards, 

PLOS ONE Editorial Office Staff

on behalf of

Dr. Abel Chemura 

Academic Editor

PLOS ONE
